# Pharmacological Properties of Chalcones: A Review of Preclinical Including Molecular Mechanisms and Clinical Evidence

**DOI:** 10.3389/fphar.2020.592654

**Published:** 2021-01-18

**Authors:** Bahare Salehi, Cristina Quispe, Imane Chamkhi, Nasreddine El Omari, Abdelaali Balahbib, Javad Sharifi-Rad, Abdelhakim Bouyahya, Muhammad Akram, Mehwish Iqbal, Anca Oana Docea, Constantin Caruntu, Gerardo Leyva-Gómez, Abhijit Dey, Miquel Martorell, Daniela Calina, Víctor López, Francisco Les

**Affiliations:** ^1^ Medical Ethics and Law Research Center, Shahid Beheshti University of Medical Sciences, Tehran, Iran; ^2^ Facultad de Ciencias de La Salud, Universidad Arturo Prat, Iquique, Chile; ^3^ Faculty of Sciences, Mohammed V University of Rabat, Rabat, Morocco; ^4^ Laboratory of Plant-Microbe Interactions, AgroBioSciences, Mohammed VI Polytechnic University, Ben Guerir, Morocco; ^5^ Laboratory of Histology, Embryology, and Cytogenetic, Faculty of Medicine and Pharmacy, Mohammed V University in Rabat, Rabat, Morocco; ^6^ Laboratory of Zoology and General Biology, Faculty of Sciences, Mohammed V University in Rabat, Rabat, Morocco; ^7^ Phytochemistry Research Center, Shahid Beheshti University of Medical Sciences, Tehran, Iran; ^8^ Facultad de Medicina, Universidad del Azuay, Cuenca, Ecuador; ^9^ Laboratory of Human Pathologies Biology, Department of Biology, Faculty of Sciences, and Genomic Center of Human Pathologies, Faculty of Medicine and Pharmacy, Mohammed V University Rabat, Rabat, Morocco; ^10^ Department of Eastern Medicine, Government College University, Faisalabad, Pakistan; ^11^ Institute of Health Management, Dow University of Health Sciences, Karachi, Pakistan; ^12^ Department of Toxicology, University of Medicine and Pharmacy of Craiova, Craiova, Romania; ^13^ Department of Physiology, “Carol Davila” University of Medicine and Pharmacy, Bucharest, Romania; ^14^ Department of Dermatology, “Prof. N.C. Paulescu” National Institute of Diabetes, Nutrition, and Metabolic Diseases, Bucharest, Romania; ^15^ Departamento De Farmacia, Facultad De Química, Universidad Nacional Autónoma De México, Ciudad De México, Mexico; ^16^ Department of Life Sciences, Presidency University, Kolkata, India; ^17^ Department of Nutrition and Dietetics, Faculty of Pharmacy, and Centre for Healthy Living, University of Concepción, Concepción, Chile; ^18^ Unidad De Desarrollo Tecnológico, UDT, Universidad De Concepción, Concepción, Chile; ^19^ Department of Clinical Pharmacy, University of Medicine and Pharmacy of Craiova, Craiova, Romania; ^20^ Department of Pharmacy, Faculty of Health Sciences, Universidad San Jorge, Zaragoza, Spain; ^21^ Instituto Agroalimentario De Aragón-IA2 CITA-Universidad De Zaragoza, Zaragoza, Spain

**Keywords:** chalcones, flavonoids, bioavailability, pharmacological studies, molecular mechanisms, clinical trials

## Abstract

Chalcones are among the leading bioactive flavonoids with a therapeutic potential implicated to an array of bioactivities investigated by a series of preclinical and clinical studies. In this article, different scientific databases were searched to retrieve studies depicting the biological activities of chalcones and their derivatives. This review comprehensively describes preclinical studies on chalcones and their derivatives describing their immense significance as antidiabetic, anticancer, anti-inflammatory, antimicrobial, antioxidant, antiparasitic, psychoactive, and neuroprotective agents. Besides, clinical trials revealed their use in the treatment of chronic venous insufficiency, skin conditions, and cancer. Bioavailability studies on chalcones and derivatives indicate possible hindrance and improvement in relation to its nutraceutical and pharmaceutical applications. Multifaceted and complex underlying mechanisms of chalcone actions demonstrated their ability to modulate a number of cancer cell lines, to inhibit a number of pathological microorganisms and parasites, and to control a number of signaling molecules and cascades related to disease modification. Clinical studies on chalcones revealed general absence of adverse effects besides reducing the clinical signs and symptoms with decent bioavailability. Further studies are needed to elucidate their structure activity, toxicity concerns, cellular basis of mode of action, and interactions with other molecules.

## Introduction

Chalcones are among the leading categories of flavonoids across the entire kingdom of plant (Hideo and Tatsurou, 1997; Abbas et al., 2014). The term chalcone is originated from the Greek name chalcos which means bronze. Chalcones were initially manufactured in the research lab in late 1800s (Shimokoriyama, 1962). The chalcone chemistry has created thorough scientific research all the way through the globe (Hideo and Tatsurou, 1997).

Naturally existing chalcones were not separated till the year 1910 (Shimokoriyama, 1962). Chalcones that derived from nature exist mostly as colors of petal and furthermore have been established in the heartwood, leaf, bark, fruit, and root of a range of plants and botanicals (Schroder, 1999).

**FIGURE 1:**
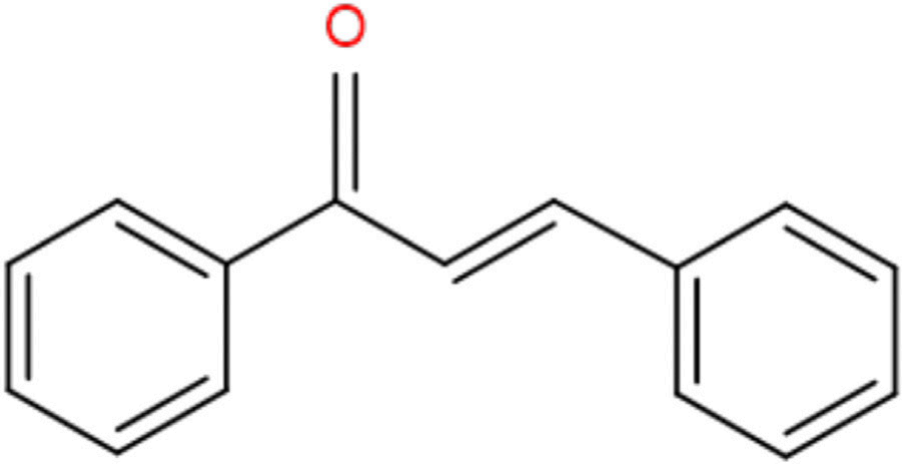
General chemical structure of chalcones.

Chalcones are also recognized as benzyl acetophenone. Chalcones are alpha, beta unsaturated ketones holding two fragrant rings (rings A and B) having different arrangement of substituents. In chalcones, two fragrant rings are connected by an aliphatic three carbon series (Rojas et al., 2002) (Figure 1).

Plants containing chalcones, for instance, the *Glycyrrhiza*, *Piper*, *Angelica*, and *Ruscus* genus, have long been utilized as therapeutic remedies in Balkan countries (Schroder, 1999; Chatzopoulou et al., 2013; Maccari and Ottana, 2015). Numerous unadulterated chalcones were accepted for clinical applications or experimented in humans. Licochalcones segregated from the plant of licorice has been stated to have a range of biological activities, for instance, antispasmodic, chemopreventive, antimalarial, antitumour, anti-inflammatory, antifungal, antioxidant, and antibacterial activities (Real, 1967; Takahashi et al., 1998). Both apples and sour fruits are loaded nutritional sources of dihydrochalcones and chalcones. Moreover, these complexes could even compose a better contribution to the overall daily consumption of unrefined or organic polyphenolics compounds than other considerably researched flavonoids (Tomás-Barberán and Clifford, 2000).

The purpose of this review is to summarize the most important pharmacological activities highlighting the cellular and molecular mechanisms of action of natural and synthetic chalcones, to better understand their therapeutic potential in the future.

## Methodology

### Search Strategy

An extensive research was conducted into the available scientific databases PubMed, Scopus, Scielo, and Science Direct using the terms “chalcones,” “bioavailability,” “biological activities,” “anti-inflammatory,” “antidiabetic,” “neuroprotective,” “antioxidant,” “anticancer,” “antibacterial,” and “antifungal.”

### Inclusion Criteria

The inclusion criteria included research studies or reviews that reported the pharmacological actions of chalcones were included; articles published in English, book chapters that also included phytochemical data, and preclinical studies on cell cultures or animal model with evidence of cellular and molecular mechanisms of action; studies that included chalcones and their derivatives from plants whose nomenclature is included in the Plant List (http://www.theplantlist.org/).

### Exclusion Criteria

The exclusion criteria included abstracts, case reports, and conference proceedings that did not meet the inclusion criteria, as well as studies that included homeopathic preparations.

### Data Collection

Selected pharmacological studies included data on chalcones and their derivatives analyzed, experimental model (*in vivo* or *in vitro*), dose, concentration, and results of pharmacological activities with molecular mechanisms included. All information obtained and analyzed in this comprehensive and updated review were summarized in tables and figures.

## Preclinical Pharmacological Activities of Chalcones

**FIGURE 2:**
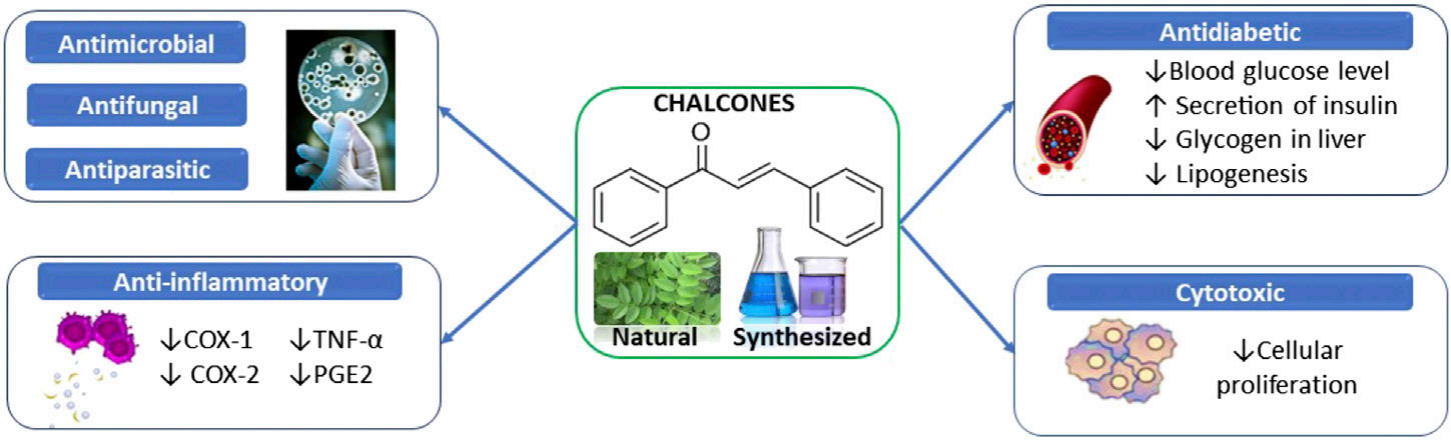
Summarized scheme of the most important pharmacological properties of chalcones.

Preclinical studies on chalcones and their derivatives have shown their high potential as antidiabetic, anticancer, anti-inflammatory, antimicrobial, antioxidant, antiparasitic, psychoactive, and neuroprotective agents (Figure 2).

### Antidiabetic Activity

#### *In Vitro* Antidiabetic Activity

Several synthetic chalcones have been reported to have potential inhibitory activity against α-glucosidase or α-amylase.

The IC_50_ value of synthetic intermediate chalcones (1–24) varied between 15 ± 0.14 and 385 ± 5.60 μM (Ansari et al., 2005). Similar observations were noted with the Chana series (Bak et al., 2011), and with the tris-chalcone derivatives (5a-5i), all showing higher inhibition profiles than those of acarbose (Burmaoglu et al., 2019). Studies on hydroxyl chalcones and bis-chalcones (1a-1m) and (2a-2m) were performed in connection to the inhibition kinetics (Cai et al., 2017). By using the abovementioned methods, natural chalcone derivatives (morusalbins A-D) showed significant inhibitory activities against α-glucosidase (Ha et al., 2018). 3ʹ,5ʹ-digeranylated chalcone (16) demonstrated noncompetitive inhibition characteristics (Ryu et al., 2010; Sun et al., 2015). In another study, compound 4m was found to be the most active compared to the other chalcone-triazole derivatives (Chinthala et al., 2015). Numerous studies have shown some chalcones and/or their derivatives (such as chalcone 1 with an IC_50_ of 840 ± 2.50 μM while that of acarbose was 860.23 ± 6.10 μM) with significant inhibitory effects than those of the standards used (Imran et al., 2015; Monisha et al., 2018).

Chalcone units of conjugates also exhibited moderate inhibitory activities against α-glucosidase (Tang et al., 2014), with the highest activity (IC_50_ = 3.2 ± 0.2 µM) recorded by conjugate 1b. Moreover, moderate inhibitory effect was observed by piperonal chalcones derivatives against α-amylase (Acharjee et al., 2018).

Four chalcone derivatives were synthesized, and it was found that the compound 3-(4-hydroxyphenyl)-1-phenylprop-2-en-1-one has an inhibitory effect on α-amylase (Attarde et al., 2014). Chalcone 4 (butein) has been shown to be the most potent compound among 41 derivatives, exhibiting significant inhibition of α-glucosidase, moderate inhibition of α-amylase, and competitive inhibition of both the enzymes (Rocha et al., 2019).

In another study, chalcone 20 was the most active inhibitor (IC_50_ = 0.4 µM) of α-glucosidase among 20 derivatives, exhibiting noncompetitive inhibition (Seo et al., 2005; Tajuddeen et al., 2018). In addition, the inhibitory capacity of chalcones 1–13 and bis-chalcones 14–18 against α-amylase (IC_50_ = 1.25 ± 1.05–2.40 ± 0.09 µM) was found to be comparable to that of acarbose (IC_50_ = 1.04 ± 0.3 µM) (Attarde et al., 2014). Furthermore, researchers have recorded promising activities of different chalcones in inhibiting the aforementioned enzymes, occupying the active sites (Najafian et al., 2010; Rawat et al., 2011; Gomes et al., 2017).

A study evaluated the antidiabetic activity of sulfonamide chalcone derivatives *in silico* using methods like homology modeled structure, molecular docking, and MD simulation. This study indicated that these derivatives can bind to residues of the active site as the same way as drugs such as acarbose and voglibose (Bharatham et al., 2008).

Prenylated chalcones (3, 4, 7) and flavanone-coupled chalcones (9, 12, 13) of *Boesenbergia rotunda* (L.) Mansf*.* roots exhibited inhibition greater than 90% at the concentration of 20 μg/ml plus an inhibitory power of α-glucosidase higher than that of acarbose (IC_50_ = 1.2 mM) (Chatsumpun et al., 2017). A natural chalcone (lavandulylated chalcone) exhibited inhibitory activity against β-glucosidase (IC_50_ = 57 μM) while noncompetitively inhibiting α-glucosidase (Kim et al., 2006). Similarly, another study isolated xanthohumol (XN) from *Humulus lupulus* L. as a potential inhibitor of α-glucosidase (IC_50_ = 8.8 μM) reversibly and noncompetitively (Liu et al., 2014). Other natural chalcones (6, 7, 20) were identified by from *Derris indica* (Lam.) Bennet root extract as a moderate inhibitor of α-glucosidase, and compound 6 showed the most potent activity (IC_50_ = 103.5 µM) (Rawat et al., 2011).

Natural prenylchalconaringenins (1) and (2) have been investigated for their inhibitory properties against digestive enzymes; 3′-geranylchalconaringenin (2) showed moderate inhibition of α-amylase (IC_50_ = 20.46 µM) and competitive and irreversible inhibition of α-glucosidase (IC_50_ = 1.08 µM) (Sun et al., 2017). In addition, these two enzymes were also inhibited by three natural chalcones from *Psoralea corylifolia* (Mounika, 2015). Another chalcone (2ʹ,4ʹ-dihydroxy-6ʹ-methoxy-3ʹ,5ʹ-dimethylchalcone) (DMC) from *Cleistocalyx operculatus* (Roxb.) Merr. and L.M.Perry flower buds inhibited pancreatic α-amylase (IC_50_ = 69.35) (Zhang and Lu, 2012). Regarding GLUT4-dependent glucose uptake, 4-hydroxyderricin (4HD) and xanthoangelol (XAG), two natural chalcones from *Angelica keiskei* (Miq.) Koidz. stem juice, increased this uptake *via* the signaling pathway of LKB1/AMP-activated protein kinase in 3T3-L1 adipocytes (Ohta et al., 2015).

#### *In Vivo* Antidiabetic Activity

Several authors have evaluated the antihyperglycemic activity of synthetic chalcones in streptozotocin-induced diabetic rats (Satyanarayana et al., 2004; Shukla et al., 2007; Najafian et al., 2010; Rawat et al., 2011; Mahapatra et al., 2017a; Sengupta et al., 2017; Shukla et al., 2017; Tajammal et al., 2017; Acharjee et al., 2018; Naidu, 2018; Raju et al., 2018). It was found that these compounds have a moderate to potential ability to reduce blood sugar. The same effect was noted in starch-loaded rats, using chalcone derivative 8c (Rawat et al., 2011). Moreover, serum glucose levels were measured in hyperglycemic rats treated with chalcone analogs, which showed a significant antihyperglycemic effect (Alberton et al., 2008).

In a study conducted by Damazio et al., it was evaluated the antihyperglycemic activity of nitrochalcones (Damazio et al., 2009) and naphthylchalcones (Damazio et al., 2010) in diabetic rats by determining blood glucose levels, insulin secretion, and 14C-glucose uptake into the soleus muscle of the animal. This indicates that the effect of chalcones on lowering blood glucose in the hyperglycemic rat can be attributed mainly to insulin secretion with potency similar to that of glipizide. In addition, the glycogen levels in the liver, brain, and spinal cord of rats were estimated following 25 mg/kg dose of chalcone administration for 7 days to discover that these chalcones were able to reduce the glycogen content in the liver, and therefore exerted a strong antidiabetic activity (Jamal et al., 2009). Furthermore, when 2-hydroxychalcone was administered to male rats, they rendered insulin resistance by a high fructose diet. This chalcone was found to have significant hypoglycemic activity by increasing insulin secretion and glycosylated hemoglobin (Jayanthi et al., 2012).

Chalcone derivatives (4A-4E) were tested on sucrose-loaded diabetic albino mice to find that compound 4-C (2-(3-(4-methoxyphenyl)-1H-pyrazol-5-yl) phenol) achieved the most promising activity, which is supported by docking study (Jain and Jain, 2017). For male mice (type 2 diabetes), at doses of 200–300 mg/kg/day, 2′, 4′-dihydroxy-4-methoxydihydrochalcone (DMC-2) exhibited a hypoglycemic effect comparable to that of metformin (antidiabetic drug) (Ribnicky et al., 2009).

Chalcone derivatives (13a-h) and (19a-h) instreptozotocin-induced diabetic mice, compounds13e, 13g, and 19f reduced TG, TC, and Glu levels, respectively (Zhu et al., 2018). Diabetic mice were treated with trihydroxychalcone derivatives, and therefore, chalcone 13 stimulated activation of AMP-activated protein kinase (AMPK), increased muscle FAO, improved tolerance to glucose, and decreased fat accumulation in the liver and skeletal muscles (Shin et al., 2018). Hypoglycemic activity of sulfonylurea chalcones 1-3 was also exhibited in normoglycemic rabbits to show that all these chalcones have activity comparable to that of gliclazide (Rao et al., 2014).

Significant hypoglycemic effects were displayed by five isoliquiritigenin (ISL) derivatives isolated from *Glycyrrhiza glabra* L. rhizomes tested in streptozotocin-induced diabetic mice (Gaur et al., 2014), chalcone-6ʹ-hydroxy-2ʹ,3,4-trimethoxy-4ʹ-O-β-D-glucopyranoside (1) from *Pouzolzia rugulosa* (Wedd.) Acharya & Kravtsova. leaves tested in alloxan-induced diabetic mice (Semwal et al., 2009), and 2′4-dihydroxy chalcone-4-glucoside from *Adhatoda zeylanica* Medik. flower (Purnima et al., 2012). Likewise, in mice with hyperglycemia, xanthoangelol (XA) and 4-hydroxyderricin (4HD), two major types of chalcones derived from *Angelica keiskei* (Miq.) Koidz*.* lowered blood sugar by demonstrating insulin-like activity with preventive effects of (4HD) on the development of diabetes in genetically diabetic KK-A^y^ mice (Enoki et al., 2007; Enoki et al., 2010).

Table 1 summarizes the *in vitro* and *in vivo* antidiabetic properties of natural and synthetic chalcones.

**TABLE 1:** Antidiabetic activities of chalcones: *in vitro* and *in vivo* preclinical pharmacological studies.

Chalcones/source	Experimental model/method	Type of study	Results/mechanisms	Ref
1-{3-[3-(substituted phenyl) prop-2-enoyl] phenyl} thioureas/synthesized	STZ-induced diabetic rats	*In vivo*	Anti-hyperglycemic: ↓blood glucose level normalization of serum biochemical parameters 10–20 mg/kg, bw	(Acharjee et al., 2018)
Intermediate chalcones 1–24/synthesized	α-Glucosidase inhibitory assay	*In vitro*	↓α-glucosidase IC_50_ = 15 mg/ml	(Ansari et al., 2005)
Chalcone derivatives (MVC1-MVC5)/synthesized	Glucose uptake in yeast cells	*In vitro*	Chalcones MCV4, MCV5: ↑ glucose uptake IC_50_ = 5–15 mg/ml	(Asogan and Aupati, 2016)
Chalcone derivatives/synthesized	STZ-induced diabetic rats	*In vivo*	Anti-hyperglycemic: ↓blood glucose level 10 mg/kg bw	(Alberton et al., 2008)
Chana chalcone derivatives/synthesized	α-Glucosidase assay dipeptidyl peptidase-4 Adipocyte differentiation	*In vitro*	Chana 1: ↓α-glucosidase, ↓DPP-4 ↑adipocyte differentiation IC_5_ = 250 μM/L	(Bak et al., 2011)
Fluoro-substituted tris-chalcones derivatives (5a-5i)/synthesized	α-Glucosidase inhibitory assay	*In vitro*	Chalcones 5a-5i: ↓α-glycosidase IC_50_ = 22.5 μM	(Burmaoglu et al., 2019)
Hydroxyl chalcones and bis-chalcones (1a-1m) and (2a-2m)/synthesized	α-Glucosidase assay Kinetics of enzyme inhibition Glucose level	*In vitro*	↓α-glucosidase Chalcones 2c, 2g, 2j,2l, are noncompetitive inhibitors Chalcone2g: ↓blood glucose level	(Cai et al., 2017)
Prenylated chalcones (3, 4, 7) Flavanone-coupled chalcones (9, 12, 13)/natural from *Boesenbergia rotunda* (L.) mansf	α-Glucosidase inhibitory assay	*In vitro*	↓α-glucosidase, IC_50_ = 1.2–20 μg/ml	(Chatsumpun et al., 2017)
Chalcone-triazole derivatives/synthesized	α-Glucosidase inhibitory assay	*In vitro*	The most active chalcones: 4m, IC_50_ = 67.78 μM 4p, IC_50_ = 74.94 μM 4s, IC_50_ = 102.10 μM	(Chinthala et al., 2015)
Chalcone derivatives/Synthesized	STZ-induced diabetic rats	*In vivo*	↑ secretion of insulin No effects on glucose uptake into muscle No effects on blood glucose levels 50 mg/kg bw	(Damazio et al., 2009)
Naphthylchalcones/synthesized	STZ-induced diabetic rats	*In vivo*	↑glucose tolerance curve ↑ secretion of insulin 10 mg/kg bw	(Damazio et al., 2010)
Xanthoangelol (XA) and 4-hydroxyderricin (4HD)/natural from *Angelica keiskei* (miq.) koidz	STZ-induced diabetic Mice	*In vivo*	Chalcone 4HD: ↓blood sugar level No effects on secretion of insulin diet containing 0.15% chalcone 4HD	(Enoki et al., 2010)
Five derivatives from isoliquiritigenin (ISL)/natural from *Glycyrrhiza glabra* L	STZ-induced diabetic Mice	*In vivo*	Anti-hyperglycemic: ↓blood glucose level 100 mg/kg bw	(Gaur et al., 2014)
Chalcone derivatives: four DAs (morusalbins A−D)/natural from *Morus alba* L.	α-Glucosidase inhibitory assay	*In vitro*	DAs (1–4, 6–8, 11, 12, 14), DAs (4, 6–8): ↓α-glucosidase IC_50_ = 2.25–5.90 μM	(Ha et al., 2018)
Chalcone 1/synthesized	α-Glucosidase inhibitory assay	*In vitro*	↓α-glucosidase, IC_50_ = 840 μM, compared with acarbose IC_50_ = 860.25 ± 6.20 μM	(Imran et al., 2015)
Chalcones: BUT, ISL, DHC, HDMC, DCC, DCCP, CMC, CMCP/synthesized	STZ-induced diabetic rats	*In vivo*	↓glycogen content in liver 25 mg/kg bw	(Jamal et al., 2009)
2- hydroxychalcone/synthesized	HFD-induced diabetic rats	*In vivo*	↓secretion of insulin ↑glycosylated hb, ↑ glucose blood level 25 mg/kg bw	(Jayanthi et al., 2012)
Lavandulylated chalcone/natural from *Sophora flavescens* aiton	α-Glucosidase β-amylase β-galactosidase α-amylase inhibitory assays	*In vitro*	↓β-galactosidase, IC_50_ = 57 μM ↓α-glucosidase, noncompetitive inhibition ↓β-amylase, mixed inhibition IC_50_ = 57 μM	(Kim et al., 2006)
Xanthohumol (XN)/natural from *Humulus lupulus* L	α-Glucosidase inhibitory assay	*In vitro*	↓ α-glucosidase; reversible, noncompetitive IC_50_ = 8.8 μM	(Liu et al., 2014)
Chalcone derivatives/synthesized	α-Amylase α-glucosidase inhibitory assays	*In vitro*	↓α-amylase, ↓α-Glucosidase IC_50_ = 1250 μg/ml	(Monisha et al., 2018)
Diarylsulfonylurea-chalcone hybrids/synthesized	STZ-induced diabetic rats	*In vivo*	Anti-hyperglycemic: ↓blood glucose level 10, 30, 50 mg/kg bw	(Naidu, 2018)
*Trans*-chalcone (benzylideneacetophenone)	STZ-induced diabetic Rats	*In vivo*	Anti-hyperglycemic: ↓blood glucose level ↑ moderate secretion of insulin 2, 8, 16, 32 mg/kg bw	(Najafian et al., 2010)
4-Hydroxyderricin (4HD) xanthoangelol (XAG)/natural from *Angelica keiskei* (miq.) koidz	3T3-L1 adipocytes	*In vitro*	Chalcones 4HD, XAG: ↑glucose uptake GLUT4-dependent through the LKB1/AMPK signaling pathway IC_50_ = 20 μmol/L	(Ohta et al., 2015)
Chalcones AC1-AC11, BC1- BC6) 2′, 4-dihydroxy chalcone -4-glucoside/synthesized and natural from *Justicia adhatoda* L	Measuring the glucose diffusion	*In vitro*	All chalcones: Good anti-hyperglycemic effect AC6: The highest activity IC_50_ = 100 μg/ml	(Purnima et al., 2012)
Chalcones (6, 7, 20)/natural from *Derris indica* (lam.) bennet	α-Glucosidase inhibitory assay	*In vitro*	↓ α-glucosidase chalcone 6: IC_50_ = 103.5 μM	(Romagnoli et al., 2008)
Sulfonylurea chalcones 1–3/synthesized	Normoglycemic rabbits	*In vivo*	All compounds: Hypoglycemic activity Compound-3: The highest activity (38.73%) 5 mg/kg bw	(Rao et al., 2014)
30-C-b-dglucopyranosyldihydro chalcone (22)/synthesized	STZ-induced diabetic rats	*In vivo*	Chalcone 22: ↓blood glucose (comparable to metformin), 25 mg/kg bw	(Rawat et al., 2011)
2′, 4′- dihydroxy-4-methoxydihydrochalcone (DMC-2)/synthesized	HFD obese C57BL/6J male mice	*In vivo*	↓blood glucose (comparable to metformin) 200–300 mg/kg bw	(Ribnicky et al., 2009)
Chalcones (1–4)/natural from *Broussonetia papyrifera* (L.) L’Hér. Ex vent	α-Glucosidase inhibitory assay	*In vitro*	Chalcones 1: ↓α-glucosidase, IC_50_ = 5.3 μM Chalcones 2: ↓α-glucosidase, IC_50_ = 11.1 μM	(Ryu et al., 2010)
Chalcones (5a-r), (4a-e), (3a-e)/synthesized	HFD sucrose STZ-induced diabetic rats	*In vivo*	Chalcones 5a, g, m, o, p, r Anti-hyperglycemic: ↓blood glucose level 100 mg/kg bw	(Satyanarayana et al., 2004)
Chalcone-6ʹ-hydroxy-2ʹ,3,4-trimethoxy-4ʹ-O-β-D-glucopyranoside (1)/natural from *Pouzolzia rugulosa* (wedd.) acharya and kravtsova	Alloxan-induced diabetic mice	*In vivo*	Hypoglycemic activity 100, 200, 500 mg/kg bw	(Semwal et al., 2009)
1-{4-[(2E)-3-(substituted phenyl) prop-2- enoyl] phenyl}-3-(substituted phenyl”) urea (2a-d), 3(a-c)/synthesized	STZ-induced diabetic Rats	*In vivo*	Anti-hyperglycemic: ↓blood glucose level doses of compounds 2(a-d) and (a-c) 35 mg/kg bw	(Sengupta et al., 2017)
Chalcone derivatives (1–20)/synthesized	α-Amylase, α-glucosidase β-amylase inhibitory assays	*In vitro*	Chalcone 20: ↓α-glucosidase IC_50_ = 0.4 μM, non-competitive inhibition	(Seo et al., 2005)
Trihydroxychalcone derivatives/synthesized	C2C12 myotubes cells HFD diabetic C57BL/6 mice	*In vitro In vivo*	Chalcone 13: ↑AMPK→ ↑ AMP-activated C_50_ = 10 μmol/L protein kinase; ↑glucose tolerance, ↑ muscle FAO, ↓fat in skeletal muscles, liver 30 mg/kg bw	(Shin et al., 2018)
Chalcone-based aryloxypropanolamines (5a-n)/synthesized	HFD sucrose and STZ-induced diabetic rats	*In vivo*	Anti-hyperglycemic: ↓blood glucose level	(Shukla et al., 2007)
Chalcone-based aryloxy-propanolamines3, 9(a, b), 10/synthesized	HFD sucrose and STZ-induced diabetic rats	*In vivo*	Chalcone 9a: ↑glucose tolerance in sucrose HFD sucrose feeded rats Chalcones 3, 9a, 9b: ↑ postprandial hyperglycaemia in STZ-induced diabetic rats 100 mg/kg bw	(Shukla et al., 2017)
3′, 5′-digeranylated chalcone (16)/synthesized	α-Glucosidase inhibitory assay	*In vitro*	↓α-glucosidase, interaction chalcone 16 and α-glucosidase’s IC_50_ = 0.90 μM	(Sun et al., 2015)
Prenylchalconaringenins (1) and (2)/natural	α-Amylase, α-glucosidase inhibitory assays STZ-induced diabetic mice	*In vitro In vivo*	3′-Geranylchalconaringenin (2) ↓α-amylase, IC_50_ = 20.46 μM ↓ α-glucosidase, IC_50_ = 1.08 μM ↓postprandial blood glucose, ↓TG, ↓cholesterol 60 mg/kg bw	(Sun et al., 2017)
Chalcones (2a, 2b, 2c)/synthesized	STZ-induced diabetic Rats	*In vivo*	Chalcone 2a: ↓blood glucose level, anti-hyperglycemic in diabetic rats Chalcone 2c: ↓blood glucose level in normoglycemic rats 100 mg/kg bw	(Tajammal et al., 2017)
Chalcone units of conjugates/synthesized	α-Glucosidase inhibitory assay	*In vitro*	All chalcones: ↓α-glucosidase Chalcone 1b:↑ inhibitory activity IC_50_ = 3.2 μM	(Tang et al., 2014)
2ʹ,4ʹ-dihydroxy-6ʹ-methoxy-3ʹ,5ʹ-dimethylchalcone (DMC)/natural from *Cleistocalyx operculatus* (roxb.) merr. and L.M.Perry	α-Amylase inhibitory assay	*In vitro*	DMC: ↓ pancreatic α-amylase IC_50_ = 69 μM	(Zhang and Lu, 2012)
Chalcone derivatives (13a-h), (19a-h)/synthesized	STZ-induced diabetic Mice	*In vivo*	Chalcones 13e, 13g, 19f; ↓TG, ↓TC, ↓Glu Chalcones 13e,19f: ↑AMPK, ↑PPARα 50 mg/kg bw	(Zhu et al., 2018)

Abbreviations and symbols: ↑, increased; ↓, decreased; STZ, streptozotocin; MD, molecular dynamic simulations; HFD, high fructose diet; GLUT-4, glucose transporter type 4; LKB1, liver kinase B1; AMPK, AMP-activated protein kinase; PPARα, peroxisome proliferator-activated receptors; BW, body weight.

### Anti-Inflammatory Activity

Literature reported several chalcones and their derivative that have shown promise to inhibit cyclooxygenase (COX) (Table 2) (Araico et al., 2006; Nyandoro et al., 2012; Bano et al., 2013; Jantan et al., 2014; Özdemir et al., 2015; Okuda-Tanino et al., 2017; Farzaneh et al., 2018). In a study to assess the anti-inflammatory effect, new chalcone derivatives using carrageenan-induced hind paw edema model, the results showed that 5′-chloro-2′-hydroxy- 4′6′-dimethyl-3, 4, 5-trimethoxychalcone (1) exhibited the most potent anti-inflammatory activity with a 90% inhibition of edema (Bano et al., 2013). In another study, a novel class indole-based chalcones were evaluated for their inhibitory effects on COX-1 and COX-2, and showed remarkable inhibition of COX-1 (Özdemir et al., 2015). The nitrogen-containing chalcone derivatives showed inhibition of some enzymes implicated to inflammatory process such as β-glucuronidase, COX-2, and trypsin (Bandgar et al., 2010). In another investigation, the synthetic fluoro-hydroxy substituted pyrazole chalcones demonstrated that exhibited selective inhibitory effect against COX-2 enzyme and a moderate effect against COX-1. The activity was related to the inhibition of COX-2 (Jadhav et al., 2013).

**TABLE 2:** Anti-inflammatory activities of chalcones.

Chalcones/source	Mechanism	Results	Ref
5′-Chloro-2′-hydroxy-4′6′-dimethyl-3, 4, 5 -Trimethoxy-chalcone/synthesized	↓ COX-1 ↓ COX-2 ↓ TNF-α	IC_50_ = 87.6 µM IC_50_ = 88.0 µM IC_50_ = 5–10 µM	(Bano et al., 2013)
3-(5-bromo-1H-indol-3-yl)-1-(4-cyanophenyl) prop-2-en-1-one/synthesized	↓ COX-1 ↓ COX-2	IC_50_ = 23.2 ± 0.5 μg/ml IC_50_ = 27.1 ± 2.5 μg/ml	(Özdemir et al., 2015)

(5-Methoxy-1H-indol-3-yl)-1-(4-(methylsulfonyl) phenyl) prop-2-en-1-one/synthesized	↓ COX-1	IC_50_ = 24.5 μg/ml no effect on COX-2	(Özdemir et al., 2015)

Hydroxy-3,4,6-trimethoxychalcone/natural from *Toussaintia orientalis* verdc	↓ COX-1	IC_50_ = 9565 μg/ml no effect on COX-2	(Nyandoro et al., 2012)

Licochalcone A/natural from *Glycyrrhiza inflata* batalin	↓ COX-1 ↓ COX-2	IC_50_ = 0.94 μg/ml IC_50_ = 1.93 μg/ml	(Okuda-Tanino et al., 2017)
(E)-3-(4-((ethylamino)methyl)-phenyl) -1-(5-methylfuran-2-yl)prop-2-en-1-one/synthesized	↓ COX-1 ↓ COX-2	IC_50_ = 25.85 μg/ml IC_50_ = 10.08 μg/ml	(Jantan et al., 2014)
Ferrocenyl-3-(4-methylsulfonylphenyl) propen-1-one/synthesized	↓ COX-2	IC_50_ = 0.05 μg/ml no effect on COX-1	(Farzaneh et al., 2018)
(E)-4-methyl-N-((4-(3-(3,4,5 trimethoxyphenyl) acryloyl)phenyl)-carbamoyl)benzenesulfonamide (Me-UCH5)/synthesized	↓ COX-2	IC_50_ = 0.06 μg/ml no effect on COX-1	(Araico et al., 2006)
(E)-1-(2,6-dimethoxyphenyl)-3-(4-(dimethylamino)phenyl)prop-2-en-1-one/synthesized	↓ PGE2	IC_50_ = 0.6 µM	(Rojas et al., 2002)
(E)-1-(2,5-dimethoxyphenyl)-3-(4-(dimethylamino)phenyl)prop-2-en-1-one/synthesized	↓ PGE2	IC_50_ = 0.7 µM	(Rojas et al., 2002)
3,4,5-Trimethoxy-4′-fluorochalcone/synthesized	↓ PGE2	IC_50_ = 0.033 µM	(Rojas et al., 2003b)
1-[6-(3,7-dimethyl-octa-2,6-dienyl)-5,7-dihydroxy-2,2-dimethyl-2H-chromen-8-yl]-3-(4-hydroxy-phenyl)- propanone/natural *Mallotus philippinensis*	↓ PGE2	IC_50_ = 7.6 µM	(Daikonya et al., 2004)
3-(3,4-dihydroxy-phenyl)-1-[6-(3,7-dime-thyl-octa-2,6-dienyl)-5,7-dihydroxy-2,2-dimethyl-2H-chromen- 8-yl]-propenone/natural *Mallotus philippinensis*	↓ PGE2	IC_50_ = 9.5 µM	(Daikonya et al., 2004)
1-[5,7-dihydroxy-2-methyl-6-(3-methyl-but-2-enyl)-2-(4-methyl-pent-3-enyl)-2H-chromen-8-yl]-3-(3,4- dihydroxy-phenyl)-propenone/natural *Mallotus philippinensis*	↓ PGE2	IC_50_ = 38.6 µM	(Daikonya et al., 2004)
Broussochalcone A/natural from *Broussonetia papyrifera* (L.) L'Hér. Ex vent	↓ PGE2	IC_50_ = 11.3 µM	(Chen et al., 2017)
Isobavachalcone/natural from *Cullen corylifolium* (L.) medik	↓ PGE2	IC_50_ = 1.6 ± 0.11 µM	(Kim et al., 2018)
Bavachromene/natural from *Cullen corylifolium* (L.) medik	↓ PGE2	IC_50_ = 2.4 ± 0.18 µM	(Kim et al., 2018)
Kanzonol B/natural from *Cullen corylifolium* (L.) medik	↓ PGE2	IC_50_ = 2.2 ± 0.21 µM	(Kim et al., 2018)
(3-(2-Hydroxyphenyl)-1-(thiophene-3-yl)prop-2-en-1-one) (TI-I-174)/synthesized	↓ PGE2	IC_50_ = 5.75 µM	(Kim et al., 2014)
2-(3-(3,4-dimethoxyphenyl)propyl)-5-methoxyphenol/synthesized	↓ PGE2	IC_50_ = 6.5 µM	(Vijaya Bhaskar Reddy et al., 2017)
(E)-1-(4-hydroxy-3-methoxyphenyl)-3-(3,4,5-trimethoxyphenyl)prop-2-en-1-one/synthesized	↓ PGE2	IC_50_ = 4.19 µM	(Hara et al., 2014)
(E)-1-(3-methoxyphenyl)-3-(3,4,5-trimethoxyphenyl)prop-2-en-1-one/synthesized	↓ PGE2	IC_50_ = 2.88 µM	(Hara et al., 2014)
2′-methoxy-3,4-dichlorochalcone/synthesized	↓ PGE2	IC_50_ = 7.1 µM	(Kim et al., 2007)
2′-hydroxy-6′-methoxychalcone/synthesized	↓ PGE2	IC_50_ = 9.6 µM	(Kim et al., 2007)
2′-hydroxy-3-bromo-6′-methoxychalcone/synthesized	↓ PGE2	IC_50_ = 7.8 µM	(Kim et al., 2007)
2′-hydroxy-4′,6′-dimethoxychalcone/synthesized	↓ PGE2	IC_50_ = 9.6 µM	(Kim et al., 2007)
2′, 5′, -dihydroxy-4-chloro-dihydrochalcone/synthesized	↓ PGE2	IC_50_ = 4.0 ± 1.5 µM	(Huang et al., 2001)
4-hydroxylonchocarpin/natural from *Psoralea corylifolia* L	↓ PGE2	IC_50_ = 10.2 µM	(Lee et al., 2005)

Natural chalcones have also shown their ability to inhibit COX-1 and COX-2: 2-hydroxy-3,4,6-trimethoxychalcone isolated from *Toussaintia orientalis* Verdc. root and stem bark extracts had a potent inhibitory effect against both the enzymes (Nyandoro et al., 2012).

Chalcones exhibited promising activity against NO and PGE2 (Table 2). The effect of dimethylamino-chalcones on the generation of NO and PGE2 mediators was studied in LPS-stimulated RAW 264.7 macrophage cells. The results showed that chalcones suppressed NO production in a dose-depending manner (Rojas et al., 2002). In another study, in order to evaluate the inhibitory effects of trimethoxychalcone derivatives on NO production, the results showed a suppression of NO and PGE2 in LPS-activated RAW 264.7 macrophage cells by 2,4,6-trimethoxy-20-trifluoromethylchalcone*.* This suggestion was supported by the data which showed an inhibition of nitrite and PGE2 levels (Rojas et al., 2003a; Rojas et al., 2003b).

Natural chalcones have also shown the ability to inhibit NO and PGE2 production. Mallotophilippen chalcones isolated from *Mallotus philippinensis* fruit extracts, exhibited suppression of NO synthesis in a murine macrophage-like cell line (Daikonya et al., 2004). Xanthohumol and dihydroxanthohumol isolated from *Humulus lupulus* L. are other natural chalcones, which considerably inhibited NO production by suppressing iNOS induced by LPS and INF-γ in a murine macrophage-like cell line (Zhao et al., 2003).

Chalcones also have proved their ability to inhibit NF-κB (Gilmore, 2006; Mahapatra et al., 2017b; Chu and Guo, 2016). Other chalcone derivatives such as isoliquiritigenin, butein, and homobutein (Orlikova et al., 2012) have suppressed TNF-α mediated by the inhibition of NF-κB gene expression (Orlikova et al., 2012). Isoliquiritigenin also reduced palmitic acid–induced macrophage activation, leading to additional anti-inflammatory activity (Watanabe et al., 2016). In human primary endothelial cells Isoliquiritigenin prevented the translocation and stimulation of NF-κB by hindering the phosphorylation and subsequent decomposition of IkBα (Kumar et al., 2007).

### Antimicrobial and Antifungal Activity

From the leaves and stems of *Crotalaria madurensis* Wight & Arn*.*, crotmadine (1) was isolated that exhibited antifungal activity (Bhakuni and Chaturvedi, 1984). Five prenylated flavonoids, including one new natural product (2–6), were isolated from an ethanol extract of the leaves of *Maclura* tinctoria (L.) D. Don ex Steud. All the isolated compounds were evaluated against *Candida albicans* and *Cryptococcus neoformans*. Compound 3 (isobavachalcone) was found to be the most active against both the yeasts (ElSohly et al., 2001). The crude methanolic extract of *Zuccagnia angulata* Hook. and Arn. by assay guided fractionation led to the isolation of two chalcones (7–8) as the compounds responsible for the antifungal activity (Svetaz et al., 2004). The antifungal activity of the chalcones (9–13), extracted from the methanol extract of the leaves of *Artocarpus nobilis* Thwaites, showed potent fungicidal activity (Jayasinghe et al., 2004). A new dimeric chalcone (14) isolated from the fresh whole uncrushed fruits of *Mallotus philippinensis* var. pallidus Airy Shaw was evaluated for antifungal susceptibility with good results (Kulkarni et al., 2014). The extracted compounds from *Zuccagnia punctata* Cav. were found to be efficacious as inhibitors of *Candida* species (Gabriela et al., 2014). In a recent study, the antifungal activity of 40 synthetized chalcones and analogs (20–59) was analyzed. Chalcones with different substituents showed to be active against different tested fungi probably by inhibiting the biosynthesis of one or both polymers of the fungal cell wall (Lopez et al., 2001). A large series of chalcones were synthesized and studied for antifungal activity against *Candida albicans*; the chalcones (60–64) exhibited promising anti-candidal activities (Batovska et al., 2007).

As part of ongoing studies in developing new antimicrobials, ten new thiazole-based chalcones (77–86) were synthesized and tested for their *in vitro* antifungal properties. These possessed modest activity against all the fungal species tested and were being less active than ketoconazole and bifonazole (Liaras et al., 2011). The chromonyl chalcones (87–88) were used as intermediates for the synthesis of new bioactive pyrazoline derivatives (89–94) under green condition. The antifungal and antimicrobial activity was tested by disk diffusion assay*.* The maximum inhibition was observed by chalcones 84 and 89 against *S. aureus* (Siddiqui et al., 2012). Using the agar cup-plate method, the antimicrobial activities of the synthesized compounds (95–106) were screened *in vitro*. The results exhibited promising antifungal activity and antibacterial activity (Prasath et al., 2013). Compound 107 was evaluated for its antibacterial properties and showed maximum zone of inhibition against *S. aureus* and *P. aeruginosa* (Bhale et al., 2013). A series of a-triazolyl chalcones were synthesized (108–121), and the synthesized compounds showed potent antibacterial activity and antifungal activity (Yin et al., 2014). A new series of pyrazine analogs of chalcones have been tested against fungal strains. The results showed that the compounds were inactive or only weekly active against most strains (Kucerova-Chlupacova et al., 2015). In another study, a series (132–179) of isatin–ferrocenyl chalcone and isatin–ferrocene conjugates were synthesized and were evaluated for their inhibitory activities against *T. vaginalis.* The compounds exhibited 100% growth inhibition (Singh et al., 2018). In another study, three chalcones, diuvaretin, uvaretin, and isouvaretin, were investigated on their antibacterial activity, and the culture inhibition was only observed for Gram-positive germs (Koudokpon et al., 2018). A series of ten chalcones and five new dihydrochromane–chalcone hybrids (189–203) were synthesized, and their antifungal activity was evaluated *in vitro*, and only two compounds had similar antifungal activity to that of the positive control (Mellado et al., 2019). A series of five fluorinated chalcones (204–208) were evaluated for their antibacterial activity against Gram-positive and Gram-negative pathogenic bacterial strains using the agar diffusion method. The results showed that the compounds exhibited broad-spectrum activity against these pathogens (Amole et al., 2019).

### Antiparasitic Activity

#### Antileishmanial Activity

The *in vitro* antileishmanial activity of chalcones was evaluated by several studies (Torres-Santos et al., 1999; Salem and Werbovetz, 2005; Salem and Werbovetz, 2006; Lima et al., 2016).

Licochalcone inhibited the growth of both *Leishmania major* and *Leishmania donovani* promastigotes and amastigotes and reduced the infection rate of human peripheral blood monocyte-derived macrophages (Chen et al., 1993). Adunchalcone displayed 50% effective concentrations against the promastigote forms of *Leishmania* (L.) *amazonensis*, *L* (V.) *braziliensis*, *L* (V.) *shawi*, and *L* (L.) *chagasi*, respectively (Dal Picolo et al., 2014). In another study, chalcones obtained *Psorothamnus polydenius* (S.Watson) Rydb*.*, and exhibited leishmanicidal properties (Salem and Werbovetz, 2005). The chalcone 2,6′-Dihydroxy-4'-methoxychalcone (DMC) showed significant activity against promastigotes and intracellular amastigotes of *Leishmania amazonensis* (Torres-Santos et al., 1999). Many other chalcone-derived plants displayed varying degrees of leishmanicidal activity such as isoliquiritigenin (Salem and Werbovetz, 2006), chalcone from *Lonchocarpus xuul* Lundell (Borges-Argaez et al., 2007), chalcones from *Calea uniflora* Less (family Compositae) (Lima et al., 2016), and sulfonamide 4-methoxychalcone derivatives (Andrighetti-Fröhner et al., 2009). A series of oxygenated chalcones demonstrated remarkable antileishmanial activity (Liu et al., 2003). The compound derived from triclosan was evaluated for antileishmanial activity against *L* (V) *panamensis* amastigotes, and the compound was found to be active against *Leishmania* parasites (Otero et al., 2014). The compounds of methoxychalcones and another synthetic chalcone, 2',4′-dihydroxychalcone displayed potent *in vitro* antileishmanial activity (Bello et al., 2011; Passalacqua et al., 2015). Also, chalcones (1–4) displayed potent leishmanicidal activity *via* reducing the infection index of macrophages significantly (De Mello et al., 2014).

*In vivo*, licochalcone A has completely prevented lesion development in *L. major*–infected mice (Chen et al., 1994; Tajuddeen et al., 2018). Chromenochalcones also showed antileishmanial potential in hamster (Gupta et al., 2014). Oral administration of chalcone 3-nitro-2-hydroxi-4,6-dimetoxychalcone (CH8) in the groups of animals infected with either *Leishmania infantum* or *Leishmania amazonensis* showed good effect (Sousa-Batista et al., 2018).

Table 3 summarizes the antileishmanial effects of chalcones using *in vitro* and *in vivo* approaches.

**TABLE 3:** Antileishmanial activity of chalcones.

Chalcones/source	Type of study	Tested effects Parasite				Ref
Licochalcone/natural	*In vitro*		*L. donovani* promastigotes amastigote form of *L. major*		IC50 = 2.4 μg/ml	(Chen et al., 1993)
2′,6′-dihydroxy-4′-methoxychalcone (DMC, 2)/natural	*In vitro*		*L. amazonensis* promastigotes	Damages of cell ultrastructure IC50 = 50 μg/ml: Damage to amastigote mitochondria IC50 = 40 μg/ml: Damage to promastigote mitochondria		(Torres-Santos et al., 1999)
Dihydrochalcones, 2′,6′-dihydroxy-4′-methoxydihydrochalcone 4/natural	*In vitro*		*L. infantum* promastigotes		IC50 = 15.30 μg/ml	(Hermoso et al., 2003)		
2',6',4-trihydroxy-4′-methoxydihydro chalcone (5)/natural	*In vitro*		*L. tropica* promastigotes *L. infantum* promastigotes		IC50 = 3.82 μg/ml IC50 = 6.35 μg/ml	(Hermoso et al., 2003)		
Chalcones from *Psorothamnus arborescens* (A.Gray) barneby/natural	*In vitro*		*L. donovani* amastigotes		IC50 = 5.0 μg/ml	(Salem and Werbovetz, 2005)		
Isoliquiritigenin/natural	*In vitro*		*L. donovani* amastigotes		IC50 = 5.30 μg/ml	(Salem and Werbovetz, 2006)		
Chalcone from *Lonchocarpus guatemalensis* benth*/*natural	*In vitro*		*L. braziliensis* promastigotes		IC50 = 10 μg/ml	(Borges-Argaez et al., 2007)		
Chalcone-triclosan hybrids/semisynthetic	*In vitro*		*L. panamensis*		IC50 = 9.4 ± 1.3 μM	(Otero et al., 2014)		
2′,4′-dihydroxychalcone 35/synthesized	*In vitro*		*L. amazonensis* promastigotes		IC50 = 0.4 μM	(Passalacqua et al., 2015)		
Methoxychalcones/synthesized	*In vitro*		*L. braziliensis* promastigote		IC50 < 10 μM	(Bello et al., 2011)		
(1E,4E)-1,5-bis(3,4,5-trimethoxy-phenyl)-penta-1,4-dien-3- one/synthesized	*In vitro*		*L. (Viannia) braziliensis*		IC50 = 1.38 ± 1.08 μM	(de Mello et al., 2014)		
(1E,4E)-1,5-bis(phenyl)-penta-1,4-dien-3-one/synthesized	*In vitro*		*L. (Viannia) braziliensis*		IC50 = 5.88 ± 1.35 μM	(de Mello et al., 2014)		
(2E)-1-phenyl-3-(3,4,5-trimethoxy-phenyl)-prop-2-en-1- one/synthesized	*In vitro*		*L. (Viannia) braziliensis*		IC50 = 6.36 ± 2.04 μM	(de Mello et al., 2014)		
(2E)-1-(4-methoxy-phenyl)-3-(3,4,5-trimethoxy-phenyl)- prop-2-en-1-one/synthesized	*In vitro*		*L. (Viannia) braziliensis*		IC50 = 5.69 ± 0.20 μM	(de Mello et al., 2014)		
Chalcone 22 Chromenochalcones/synthesized	*In vivo*		*L. donovani*/hamster model		50 mg/kg/day→↓parasites 48.53 ± 10.43% on day 7 post treatment	(Gupta et al., 2014)		
Chalcone 37 Chromenochalcones/synthesized	*In vivo*		*L. donovani*/hamster model		50 mg/kg, for10 days→ ↓parasites (83.32 ± 12.37%)	(Gupta et al., 2014)		
Chalcone-triclosan hybrids/semisynthetic	*In vitro*		*L. panamensis*		IC50 = 9.4 ± 1.4 μg/ml	(Otero et al., 2014)		

#### Antimalarial Activity

Naturally occurring chalcones have demonstrated promising potencies after being tested *in vitro* against *Plasmodium falciparum*. The compounds bartericin A, stipulin 3, 4, and hydroxylonchocarpin demonstrated particular antimalarial potential with relatively low doses (Ngameni et al., 2007). Other chalcones with antimalarial activity proved *in vitro*: cajachalcone (Ajaiyeoba et al., 2013), xanthohumol (Frölich et al., 2009), sulfonamide chalcone derivatives (Domínguez et al., 2005), sythesized novel chlorovinyl sulfone-like chalcone derivatives (Dominguez et al., 2009), quinolinyl chalcones synthesized (Domínguez et al., 2001), various 1,3-diaryl-2-propenones (chalcone derivatives) (Geyer et al., 2009), and chalcone chloroquinolines such as chloroquine and quinine (Hayat et al., 2011). Among the 27 novel chalcone derivatives synthesized, only one compound was found to be the most antimalarial active (Yadav et al., 2012).

Chalcone derivatives administrated intraperitoneally to the *Plasmodium yoelii*–infected mice model showed significant inhibition of these strains (Tomar et al., 2010).

Table 4 summarizes the principal studies carried out on the antimalarial effect of natural and synthetic chalcones.

**TABLE 4:** Antimalarial activity of chalcones.

Chalcones	Source	Method	Type of study	Parasite	Effects	Ref
Bartericin A1	Natural	Culture W2 strain of *P. falciparum*	*In vitro*	*P. falciparum*	IC_50_ = 2.15 ± 0.02 μM	(Ngameni et al., 2007)
Bartericin B2	Natural	Culture W2 strain of *P. falciparum*	*In vitro*	*P. falciparum*	IC_50_ = 19.27 ± 0.06 μM	(Ngameni et al., 2007)
Stipulin 3, 4	Natural	Culture W2 strain of *P. falciparum*	*In vitro*	*P. falciparum*	IC_50_ = 5.13 ± 0.04 μM	(Ngameni et al., 2007)
Hydroxylonchocarpin 4	Natural	Culture against the W2 strain of *P. falciparum*	*In vitro*	*P. falciparum*	IC_50_ = 3.36 ± 0.07 μM	(Ngameni et al., 2007)
Isobavachalcone 5	Natural	Culture against the W2 strain of *P. falciparum*	*In vitro*	*P. falciparum*	IC_50_ = 19.00 ± 0.02 μM	(Ngameni et al., 2007)
Kanzonol B	Natural	Culture against the W2 strain of *P. falciparum*	*In vitro*	*P. falciparum*	IC_50_ = 9.63 ± 0.04 μM	(Ngameni et al., 2007)
Cajachalcone	Natural	The bioassay-guided fractionation of methanol extract of C. cajan leaves	*In vitro*	*P. falciparum*	IC_50_ = 2.0 µg/mL	(Ajaiyeoba et al., 2013)
Xanthohumol and seven derivatives	Semi -Synthetic	—	*In vitro*	*P. falciparum*	IC_50_ = 8.4 ± 0.3 μM (poW) IC_50_ = 24.0 ± 0.7 μM (Dd2)	(Frölich et al., 2009)
Sulfonamide chalcone derivatives	Synthetic	Culture of *P. falciparum* parasites	*In vitro*	*P. falciparum*	IC_50_ > 10 μM	(Domínguez et al., 2005)
Sulfonamide chalcone derivatives	Synthetic	b-hematin formation	*In vitro*	*P. falciparum*	IC_50_ = 0.48 μM	(Domínguez et al., 2005)
Quinolinyl chalcones derivatives	Synthetic	Culture of *P. falciparum* parasites	*In vitro*	*P. falciparum*	IC_50_ = 19.0 μM	(Domínguez et al., 2001)
Hlorovinyl sulfone-like chalcone derivatives	Synthetic	Claisen–Schmidt condensation	*In vitro*	*P. falciparum*	IC_50_ = 0.025–10 mM	(Dominguez et al., 2009)
Phenylurenyl chalcone	Synthetic	-	*In vitro*	*P. falciparum*	IC_50_ = 1.76 μM	(Domínguez et al., 2005)
-(2,5-dichlorophenyl)-3-(4-quinolinyl)-2-propen-1-one	Synthetic	-	*In vitro*	*P. falciparum*	IC_50_ = 200 nM	(Li et al., 1995)
Chloroquinoline	Synthetic	Claisen–Schmidt condensation	*In vitro*	*P. falciparum*	IC_50_ = 31.54 mM	(Hayat et al., 2011)
1-(4-Benzimidazol-1-yl-phenyl)-3-(2, 4-dimethoxy-phenyl)-propen-1-one	Synthetic	Claisen–Schmidt condensation	*In vitro*	*P. falciparum*	IC_50_ = 1.1 μg/ml	(Yadav et al., 2012)
Licochalcone	Synthetic	—	*In vitro*	*P. falciparum*	IC_50_ = 1.43 μg/ml	(Yadav et al., 2012)
Acridinyl chalcone derivatives (1a–k)	Synthetic	Noncatalyzed nucleophilic aromatic	*In vitro*	*p falciparum*	IC_50_ = 2 mg/ml	(Tomar et al., 2010)
Chalcone-AZT hybrid series 7 and 9Acetylenic chalcones (1a–c, 2a–e) Chalcone-chloroquinoline hybrid compounds (8 and 10)	Synthetic -	—	*In vitro*	*p falciparum*	Compound 8b was the most active, submicromolar IC_50_ values against the D10, Dd2 and W2 strains of *P. falciparum*.	(Guantai et al., 2010)
Alkoxylated Chalcones	Synthetic	-	*In vitro*	*P. falciparum*	IC_50_ = 6.5 mM	(Nowakowska, 2007)
4-Chloro-20,40-dihydroxychalcone	Synthetic	-	*In vitro*	*P. falciparum*	IC_50_ = 12.3 mM	(Nowakowska, 2007)
Hydroxylated chalcones	Synthetic	-	*In vitro*	*P. falciparum*	IC_50_ = 20 mM	(Nowakowska, 2007)
Phenylurenyl chalcone derivatives	Synthetic	-	*In vitro*	*P. falciparum*	IC_50_ = 1.75–10 mM	(Nowakowska, 2007)
Xanthohumol	Synthetic	-	*In vitro*	*P. falciparum*	IC_50_ = 8.2 mM IC_50_ = 24 mM	(Nowakowska, 2007)

### Cytotoxic and Antiproliferative Activity

Chalcones (natural and derivatives) displayed potent antiproliferative consequences in both initial as well as developed ovarian cell carcinoma (De et al., 1995) and also in stomach carcinoma HGC-27 cell (Shibata, 1994) (Table 5).

**TABLE 5:** Cytotoxic and antiproliferative activity of chalcones.

Chalcones	Source	Type of study	Effects	Ref.
Chalcones with piperazine moiety.	Synthetic	*In vitro* (different cancer cells)	Anticarcinogenic properties	(Filosa et al., 2007) (Rahaman et al., 2010)
Imidazoquinonyl chalcones and pyrazolines.	Synthetic	*In vitro* (HeLa cells)	Anticarcinogenic properties	(Viveka et al., 2014)
β-carboline based chalcones.	Synthetic	*In vitro* (MCF-7 cells)	DNA fragmentation and apoptosis	(Chauhan et al., 2014)
Heteroaromatic chalcones.	Synthetic	*In vitro* (T47D cells)	Topoisomerases inhibitory and cytotoxic activity	(Jeon et al., 2016)
Chalcone derived compounds replaced acetophenone and replaced aldehyde.	Synthetic	*In vitro* (MCF-7 cells)	Apoptosis induction in MCF-7 cells with the involvement of caspase-7, caspase-8, and caspase-9	(Syam et al., 2012)
Thiophene analogues of chalcones.	Synthetic	*In vitro* (K562 cells)	Inhibition of Tubulin polymerization	(Romagnoli, 2008)
Chalcone derived compounds Hsp90 inhibitors	Synthetic	*In vitro* (H1975 and MDA-MB-231 cells)	HSP90 inhibitory effect	(Jeong et al., 2014; Oh and Seo, 2017)

Chalcones with piperazine moiety have demonstrated different, as well as, crucial pharmacological activities counting antihistamine (Rahaman et al., 2010), antioxidant, anti-inflammatory (Bandgar and Gawande, 2010), anti-infective (Tomar et al., 2007), and anticarcinogenic properties (Filosa et al., 2007). In the light of piperazine moiety, biological activity has also been reported and encouraged.

Chalcones with piperazine moiety were created, and their *in vitro* anti-carcinoma–producing activity was observed (Rahaman et al., 2010). New fragrant chalcones with *in vitro* anti-carcinoma–producing property have also been recorded (Viveka et al., 2014). In addition, Jurkat cell line of human T-lymphocyte blood cancer along with HL-60 human blood cancer cell lines is also targeted by diaryl chalcones. The *in vitro* study was performed for ascertaining compound activity in opposition to two breast carcinoma cell lines MCF-7 (Chauhan et al., 2014) and T47D (Jeon et al., 2016). Table 6 The result specified that all the compounds were dynamic but not analogous with doxorubicin. However, it displayed some effects against two breast carcinoma cell lines (Ugwu et al., 2015). In another study, 25 chalcone-derived compounds were reported to exhibit anticarcinogenic properties (Syam et al., 2012). Recent research conducted on 46 different chalcones to measure exact antiproliferative activities against the human tumor necrosis factor–associated programmed cell death–inducing ligand (TRAIL) against cervical (HeLa), liver (HepG2), breast (MCF-7, MDA-MB-231), ovarian (Caov-3), nasopharyngeal (CNE-1), erythromyeloblastoid (K-562), lung (A549), colorectal (HT-29), T-lymphoblastoid carcinoma cells (CEM-SS), and common human embryonic kidney (HEK-293) cells.

**TABLE 6:** *In vitro* **s**ummarization of recent research on heteroaromatic chalcones (Jeon et al., 2016).

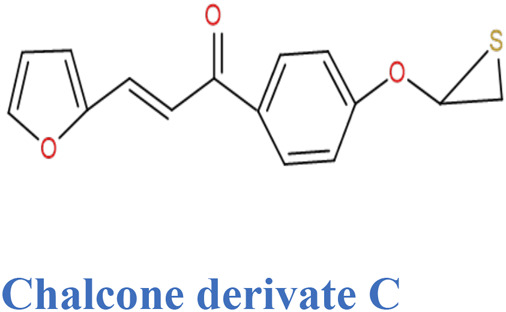	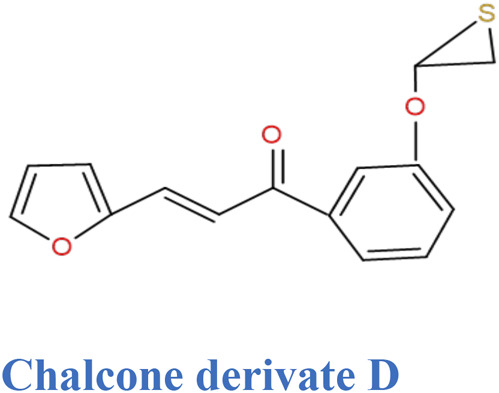	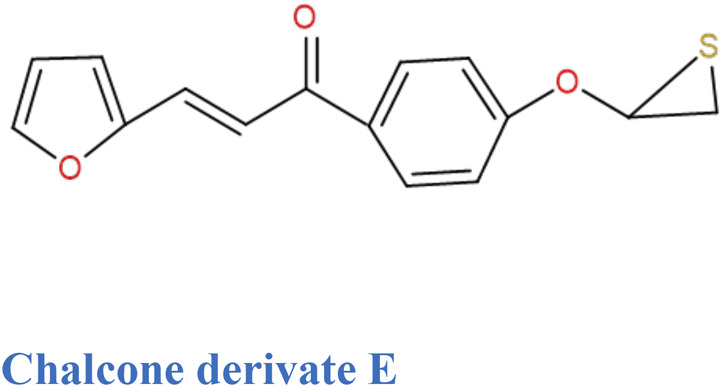	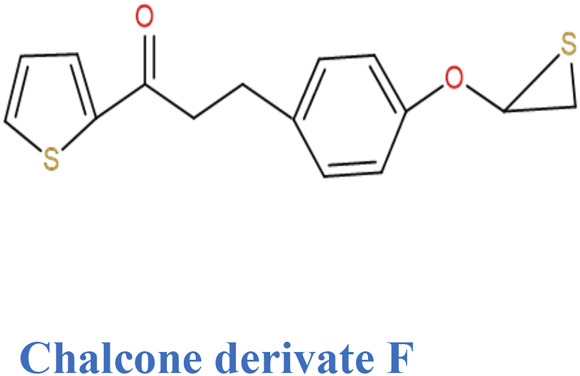
**Tested cells lines**			
MDA-MB231 basal resembling (more invasive) human triple negative breast adenocarcinoma cell line.MDA-MB468 human triple negative breast adenocarcinoma cell line originated from metastatic spot T47D human breast ductal cancer cell line			
**Results**			
IC_50_ = 100 µM ↓TI^1^ > 60%	IC_50_ = 100 µM ↓TI^1^ > 70%	IC_50_ = 100 µM ↓TI^1^ > 60%	IC50 = 100 µM ↓TI1 < 5%
↓TII^2^ > 90%	↓TII^2^ > 90%	↓TII^2^ > 90%	0% inhibition of TII
↓T47D carcinoma cells proliferation	↓activity against all cell lines comparing with others three chalcones	↑anti-proliferative activity against MDA-MB468	↓T47D carcinoma cell proliferation IC50 = 3.85 µM ↑antiproliferative effect (control camphothecin)

Chalcone derivatives with enone and thiophene rings also possess activity against tubulin assembly and colchicines; they bind to tubulin of K562 cells (chronic myeloid leukemia; CML) and inhibit their growth on G_2_/M stage of the cell cycle (Romagnoli, 2008). In addition, those thiophene chalcone derivatives inhibit human T-lymphocyte (Molt 4 and CEM) and human cervix cancer (HeLa) cells. This research was conducted on murine blood carcinoma (L1210), murine mammary cancer (FM3A), human HeLa, Molt 4, and CEM cells by taking 0.3–0.5 million cells/mL of culture medium. After incubating the cells with testing compounds at 37°C for 2 days, cell number was counted by means of a Coulter counter.

#### Anticancer Potential of Chalcones

*Cancer* is one of the most feared diseases of the 21st century—according to the 2012 Globocan report, 14 million people are diagnosed with cancer each year and more than 8 million deaths are reported each year (Ferlay et al., 2013). Because radiotherapy or chemotherapy has multiple adverse effects, new molecular therapies are being tested for use in the treatment of solid tumors and blood cancers (Ahmad Farooqi et al., 2017; Salehi et al., 2019e). Targeted molecular therapy uses the patient’s genetic information to determine which molecules can act most effectively in concerning to the type of diagnosed cancer (Zajac et al., 2016; Žiberna et al., 2017; Moosavi et al., 2018). Thus, heat-shock proteins 90 (HSP90) inhibitors open new perspectives in cancer treatment by destabilizing proteins by which cancer cells survive and multiply (tumorgenesis) (Amolins and Blagg, 2009). Recent studies from last years have shown that synthetic chalcones can have a HSP90 inhibitory effect (Jeong et al., 2014; Oh and Seo, 2017). Several phase II clinical trials of new anticancer molecules that have two hydroxyl groups at positions 1,3 revealed inhibition of interactions between HSP90 and patients' proteins through binding of these molecules to the ATP site in HSP90 (Butler et al., 2015). Maybe in the future, phase III clinical trials will be conducted to support the anticancer potential of chalcones and their derivatives.

### Neuroprotective Activity

A research has been conducted on 10 different chalcones, out of which two have nearly similar activity as of diazepam: isoliquiritigenin (ISL, 2’,4’,4-trihydroxychalcone) and butein (BUT, 2’,4’,3,4,tetrahydroxychalcone). This research based on outcomes of chalcones on different replacements, investigated in animal models for instance open field experiment, equine protozoal myeloencephalitis test, rotarod performance, and grip analysis. These experiments are typical models for screening CNS actions giving information regarding tranquilizing or sleep inducing, psychomotor performance, anxiety, and muscle-relaxant effects (Tsatsakis A. M. et al., 2019). The kinetic study of ISL to monoamine oxidase-A indicated that it merged to variable positions of the enzyme, independent of the pre-binding of serotonin (Tan et al., 2000). In the wide-ranging perception, reasonably lipophilic medicines traverse the blood–brain barrier (BBB) by submissive diffusion (Salehi et al., 2020c; Sharifi-Rad et al., 2020d). Opposing molecules are usually poor central nervous system agents, except they pass through dynamic transport across the central nervous system (Pajouhesh and Lenz, 2005; Salehi et al., 2020a). Hence, it can be approximated that they are able to traverse the BBB and attain their target (Di et al., 2003; Calina et al., 2020; Sharifi-Rad et al., 2020e). Chalcones one, nine, fourteen, fifteen, and sixteen with fine affinity for the BZD binding positions of the GABA category A receptors, chalcones one and five with attraction for the 5-hydroxytryptamine_1A_ receptor, and compounds six and twelve for the µ-opioid receptor were preferred to be experimented as antidepressants, anti-anxiety agents, and against the sensation and perception of pain in extensively applied pharmacological experiments in rats (Salehi et al., 2019c). During the tail suspension experiment, chalcone one demonstrated antidepressant-like activity in rodents, while compound six demonstrated action against sensations and perceptions of pain in an acute chemical stimulated nociception assessment.

The new fifty-methyl-twenty-hydroxy-thirty-nitrochalcone exhibited marginal and central activities against perceptions and sensations of pain either in acute thermal or chemical nociception experiments. According to the consequences recapitulated, plain chalcone derived compounds are favorable compounds for the discovery and growth of new central nervous system medicines and contain an encouraging scaffold in medical chemistry for the evolution of medicines and for the management of pain, depression, and anxiety (Dominguez et al., 2009).

## Chalcones in Clinical Trials

### Chalcones in Treatment of Chronic Venous Insufficiency

Chronic venous insufficiency (CVI) is a clinical syndrome that results from chronic disorders of venous circulation from the lower limb level. The main symptoms in moderate stages are heavy legs, tension in the lower limbs, varicose veins dilated, followed in severe stages by swelling of the lower limbs, skin changes, and the appearance of venous ulcer (Lichota et al., 2019). A therapeutic option is represented by laser therapy, sclerotherapy, and venoactive drugs (Ianosi et al., 2019). These venoactive drugs are a heterogeneous group of substances from plant or synthetic origin that modulate the venous tone, attenuates the blood rheology, improves micro- and macrocirculation, regulates capillary permeability, have anti-inflammatory effects by inhibiting leukocyte–endothelial interaction, and reduces the oxidative stress (Salehi et al., 2020b).

Recent clinical trials have shown the main role of two chalcones hesperidin methylchalcone and hesperidin trimethylchalcone in the treatment of chronic venous disorders (Boyle et al., 2003) and varices of the trunk of the internal saphenous vein, respectively (Weindorf and Schultz-Ehrenburg, 1987). In a randomized open-label study, the therapeutic effect of a mixture of hesperidin methyl chalcone, *Ruscus aculeatus* with vitamin C compared to rutozide in patients diagnosed with chronic venous insufficiency was investigated (Beltramino et al., 2000). This clinical trial was conducted for three months and included eighty patients divided into two groups: the first group received the combination with hesperidin methyl chalcone, and the second received only rutoside. The signs and symptoms of chronic venous insufficiency were evaluated initially and then monthly. From the clinical point of view, a significant and lasting reduction of the symptoms was obtained in the patients from the first group treated with the mixture of chalcone and vitamin C compared to the second group, treated only with rutozide (Beltramino et al., 1999).

The mechanism of the venotonic effect of *Ruscus* and hesperidin methylchalcone extract is exerted by a two-way adrenergic mechanism: 1) direct effect as agonist of the postjunctional alpha-adrenergic receptors of the smooth cell in the vascular wall and 2) indirect effect expressed by increasing the release of noradrenaline from the presynaptic vesicles (Beltramino et al., 1999; Peralta et al., 2007; Gomes et al., 2017). The dose–effect relationship in the single dose and the respective role of each constituent of this combination with hesperidin methylchalcone (150 mg), *Ruscus aculeatus* plant (150 mg per capsule), and ascorbic acid (100 mg) on the venous tone were also demonstrated in a clinical study that included 37 women with superficial venous insufficiency. It has been shown that the effect of a capsule administered twice daily is similar to the administration of two capsules daily in the morning, and no adverse digestive effects have been reported. Clinical efficiency consisted in improving the permeability of the vascular walls, increasing the vascular tone, reducing the edema and normalizing the blood circulation in the blood vessels (Boccalon et al., 1998). Similar beneficial effect of hesperidin methylchalcone (HMC) on lymphatic venous insufficiency in a recent meta-analysis of some clinical trials has also been demonstrated. The good tolerability and the reduced adverse effects of the combination of HMC, *Ruscus* extract, and vitamin C have led the specialists to propose their inclusion in the new treatment guidelines for chronic venous insufficiency (Kakkos et al., 2018).

In a randomized double-blind study, the pharmacological effect of trimethyl hesperidine chalcone associated with *Ruscus* extract and vitamin C was demonstrated in patients diagnosed with femoral trunk varicose (Weindorf and Schultz-Ehrenburg, 1987). The study included fifty patients, divided into two groups: one orally treated 14 days with this combination and the other with placebo. In both the groups, the venous tone was evaluated by plethysmography, both in motion and at rest. In the group treated with trimethyl hesperidine chalcone associated with *Ruscus* extract and vitamin C, the clinical signs were significantly reduced (Weindorf and Schultz-Ehrenburg, 1987).

### Chalcones in Treatment of Skin Conditions

Skin diseases are leading causes of morbidity with high prevalence and incidence, affecting the patients’quality of life and being associated with very important social, economic, and healthcare costs (Ianosi et al., 2018; Scheau et al., 2020). This is why the search for new treatment options in dermatology is one of the most important research areas in both fundamental and clinical science (Ianoşi et al., 2016; Sifaki et al., 2020).

Various clinical trials have evaluated the role of chalcones in inflammatory skin conditions and one of the most investigated substances was licochalcone A. An interesting study including sixty-two women with persistent mild to moderate facial redness (Weber et al., 2006) has evaluated skin compatibility and effect of a skin care regimen containing licochalcone A with duration of 8 weeks. The topical products were very well tolerated, and the results of the study showed significant improvements of erythema and in quality of life of the patients. A subsequent study on 33 rosacea patients showed that the skin care products with licochalcone A are compatible with the standard topical treatment of the disease.

Another research has assessed the effects on sensitive skin of licochalcone A in combination with 4-t-butylcyclohexanol (Sulzberger et al., 2016). The authors have conducted a single-blind, randomized study in order to evaluate subjective and objective symptoms of skin sensitivity. The formulation containing licochalcone A-rich licorice extract combined with 4-t-butylcyclohexanol showed a significant reduction of shaving-induced erythema. It was suggested that the anti-inflammatory effect of licochalcone A is induced by a significant reduction of NFκB signaling and prostaglandin E2 (PGE2) secretion.

A recent randomized, prospective, investigator-blinded study (Boonchai et al., 2018) has evaluated the effects of a moisturizer containing 4-t-butylcyclohexanol and licochalcone A on eighty patients with mild to moderate facial dermatitis. The chalcone containing topical treatment has induced significant improvements of clinical aspect, hydration of cutaneous tissue, and transepidermal water loss as well as the patients' subjective evaluation. The results of facial moisturizer were compared with those induced by 0.02% triamcinolone acetonide cream and even if the topical corticoid treatment was associated with faster improvement of patients’ symptoms, the chalcone containing moisturizer showed better effects on skin hydration and inflammation control.

A complex research including two clinical studies and several *in vitro* experiments was conducted in order to evaluate the anti-irritative effect of cosmetic formulations containing licochalcone A (Kolbe et al., 2006). The prospective randomized vehicle-controlled clinical trials enrolled a total of 57 healthy subjects, 45 of them being included in study using a post-shaving skin irritation model and 12 volunteers taking part in a UV-induced erythema test. Even if in one model inflammation was induced by impairment of skin barrier and in the second by UV-penetration damage, in both studies, the topically applied licochalcone A-rich licorice extract showed a highly anti-irritative effect, significantly reducing erythema. The additional *in vitro* data emphasized possible cellular and molecular mechanisms showing a strong inhibitory effect of licochalcone A on pro-inflammatory responses of different cell types such as granulocytes, keratinocytes, dermal fibroblasts, and monocyte-derived dendritic cells.

Moreover, licochalcone A has proved to be effective in scalp disorders. The effect of a tonic solution containing licochalcone A, among other active components, has been investigated in 30 subjects with dry and itchy scalp conditions and showed a significant reduction of scalp dryness, itching, and microinflammation (Schweiger et al., 2013). The role of chalcones in the treatment of inflammatory skin conditions in children is another important area of research. A randomized, double-blind, split-side comparison study on 75 infants between the age of 2 weeks and 1 year showed that a moisturizer containing 0.025% licochalcone is equally effective as topical 1% hydrocortisone for the treatment of infantile seborrhoeic dermatitis (Wananukul et al., 2013). The same research group, in a multicenter randomized, prospective, split-side, double-blind study, has evaluated the effect of a moisturizer containing licochalcone A compared to 1% hydrocortisone topical therapy in the treatment of childhood atopic dermatitis (Wananukul et al., 2013). The study included 55 children with mild to moderate lesions and showed that the moisturizer containing licochalcone A significantly reduces the clinical severity of the lesions and the transepidermal water loss, being equally effective as topical corticosteroid treatment. Moreover, continuing the treatment with licochalcone A moisturizer was able to stabilize the clinical improvement and the skin barrier recovery. These results are in accordance with data from a previous randomized, controlled, investigator-blinded study (Udompataikul and Srisatwaja, 2011).

Chalcones are also evaluated as potential treatment options in acne patients. A double-blinded, prospective, randomized, vehicle-controlled clinical trial has investigated the tolerability and effect of a moisturizer containing licochalcone A, l-carnitine, and 1,2-decanediol as adjuvant treatment in topical therapy with retinoids (Chularojanamontri et al., 2016). The study included 120 subjects with mild to moderate acne and showed a significant reduction of total lesions in patients treated with the moisturizer containing active substances. Moreover, they had less inflammatory lesions and skin irritations.

Anti-aging medicine is another important area of research in which chalcones are investigated (Sharifi-Rad et al., 2020b). A double-blind, placebo-controlled trial including ninety-two subjects showed that oral intake of *Boesenbergia pandurata* extract containing panduratin A as bioactive compound for 12 weeks significantly increases skin hydration and gloss and decreases wrinkling without any adverse symptoms, suggesting a possible use of *Boesenbergia pandurata* extract as a nutraceutical or nutricosmetic product (Kim et al., 2017).

## Bioavailability of Chalcones

Research on the bioaccessibility of chalcones from sources of food are bounded, but experimented artificial chalcones have accounted to contain broad ranges of biological activities (Won et al., 2005). Although chalcones have an essential position in the bio-production of flavonoids (Shirley, 1996) and are familiar in a number of foods and drinks, like rooibos tea or apples, there are unavailability of data on their bioaccessibility in human beings.

The prenylated chalcone xanthohumol is the amplest chalcone produced in hop cones. Throughout beer preparation, a huge fraction of xanthohumol is changed to the related isomeric prenylflavanone isoxanthohumol. Following administration of xanthohumol to rodents by force feeding at extremely elevated dosage (1 g/kg of body weight), linked metabolites were identified in plasma. The most important metabolite, xanthohumol- 4*9*-*O*-glucuronide, attained its topmost concentration of 3.1 lmol/L 4 h after administration. The maximum concentration of unmetabolized xanthohumol was 10 times lower with the similar Tmax of 4 h (Gerhäuser, 2005). One more rodent study discovered only conjugates in plasma following oral administration of xanthohumol however unsuccessful to distinguish unmetabolized xanthohumol (Avula et al., 2004).

Conversely, these studies demonstrate that prenylated chalcones are bioavailable, although their bioaccessibility appears to be commonly low.

Another study explored the prospective accessibility of flavanones in diversely processed *Citrus sinensis* (L.) Osbeck juices by imitating stomach and small intestinal *in vitro* digestion (Gil-Izquierdo et al., 2001).

In addition to showing the power of pasteurization and storage on the substance of dissolvable flavanones, these researchers detected that *in vitro* pancreatin intake of *Citrus sinensis* (L.) Osbeck juice in a mild alkaline medium, imitating absorption in the small intestine, converted fifty to sixty% of the dissolved flavanones (primarily hesperidin) to chalcones (principally hesperidin chalcone) (Cermak et al., 2009). Particularly the poor dissolvability of a large number of chalcone compounds, the bioequivalence effectiveness has not achieved the anticipated intensities in preclinical assessments.

Therefore, the maximization of the physicochemical activities will be one of the principal study routes of chalcone-dependent compounds. For the objects of chalcone compounds, a number of anticipated targets must be confirmed. Activity-dependent protein outlining is a potent approach for recognition of target that must be decided by considering each case individually because of the properties of chalcone molecules (Zhuang et al., 2017).

## Discussion

The results of our study confirmed the therapeutic potential of chalcones. The limitations of this research result from the fact that many meta-analyzes were included and not individual studies. But this can be considered as a strong point because recent meta-analyzes have summarized the most important pharmacological effects *in vitro* and especially *in vivo*. Another strength of this review is that the latest studies and clinical trials on patients have been described, thus confirming the clinical importance and positive prospects in medical therapy.

Natural and synthetic chalcones and their derivatives presented antidiabetic effects, and the effect can be attributed mainly to lowering of insulin secretion with potency similar to that of hypoglycemic agents (ig Glipizide) (Jamal et al., 2009). Numerous studies have reported the anti-inflammatory effects of chalcones on several targets such as enzymes implication in promoting inflammation process: cyclo-oxygenase, interleukins, nitric oxide synthase, cell adhesion molecules (CAM), lipo-oxygenase (LOX), and prostaglandins (PGs) (Salehi et al., 2020c; Mititelu et al., 2020). The suppression and/or inhibition of cyclo-oxygenase enzyme is a promising therapeutic way in the treatment of inflammatory diseases (Salehi et al., 2019d; Sharifi-Rad et al., 2020c). Many bioactive compounds, both natural and synthetic, have been isolated and synthetized to develop anti-cyclooxygenase activity (Salehi et al., 2019b; Padureanu et al., 2019; Sharifi-Rad et al., 2020a). PGE2 and NO are among the inflammatory mediators that promote inflammation in several diseases (Salehi et al., 2019c; Salehi et al., 2020b). Consequently, the inhibition of these mediators is strongly suggested as remedy for numerous inflammatory diseases. (Mocan et al., 2014; Tsatsakis A. et al., 2019; Toiu et al., 2019). Chalcones also have proved their ability to inhibit NF-κB (nuclear factor kappa) which regulates the most important factors involved in inflammatory process such as cytokines, chemokines, and adhesion molecules (Salehi et al., 2019d; Salehi et al., 2019a). Several studies have suggested the use of chalcones and their derivatives target specifically NF-κB as an anti-inflammatory therapeutic strategy (Chu and Guo, 2016).

Chalcones are natural products, produced by plants as a natural defense mechanism against pathogens as fungi and bacteria. Synthesized β-chlorovinyl chalcones exhibited antifungal activity (Bandgar and Gawande, 2010). In general, the natural chalcones (synthesized or modified) are being increasingly documented because of their interesting antimicrobial activities and can be represented as promising agents in the perspective of new antibiotic drugs discovery. Some of the chalcones have been implicated in inhibition of exoenzymes responsible for fungal invasion mechanisms, also inhibiting biofilm and germ tube formation as in *C. albicans*. They may affect the cellular cytoplasmic membrane and induce cell apoptosis as it was noted in case of carvacrol (Zuzarte et al., 2012). In addition, it was also reported that flavonoid compounds as chalcones inhibit the growth of bacteria by acting on the membrane potential which might affect the overall bacterial metabolic activity, resulting in some biosynthetic pathway inhibition, as demonstrated by the strong inhibition of DNA, RNA, and protein synthesis (Dzoyem et al., 2013; Ungureanu et al., 2017). Chalcones also showed to be a promising anticancer potential because it induces selective cell death in carcinoma cells with not upsetting regular cells (Syam et al., 2012) and psychoactive and neuroprotective activities. (Brady et al., 2012).

## Overall Conclusions and Future Perspectives

The curiosity and attraction toward natural compounds are increasing gradually because of the recognized favorable consequences on numerous prevalent and general diseases like carcinoma, allergic reactions, cardiovascular disease, infectious diseases, parasitic diseases, type 2 diabetes mellitus, or diseases of central nervous system. Starting from the ethnopharmacological uses of chalcones, in this study, the most important *in vitro* and *in vivo* biological activities such as antibacterial, antioxidant, antineoplastic, cytotoxic, antiulcer, antidepressant, anxiolytic, and anti-inflammatory were highlighted. Chalcones derivatives have shown anticancer activity against a variety of cancer cell lines, antibacterial activity against Gram-negative and Gram-positive germs, and anti protozoal activity. Although conducted in a small number, clinical studies of chalcones have shown a lack of adverse effects in patients with chronic venous insufficiency, the reduction of clinical signs and symptoms, and good plasma concentrations. However, further clinical studies are needed to fully understand the mechanisms of action at the cellular level and to establish correlations between their structure and pharmacological actions, especially anticancer activity.

Although they showed many interesting biological effects and many preclinical experiments could be performed, their mechanism of action is not entirely known. Being compounds that could be synthesized relatively easily, in the future, it is necessary to develop new synthesis methods that allow the research of new biological properties, a deeper knowledge of the molecular mechanisms of action, and especially the identification of the target of the action. And so, this successful story of the promising therapeutic effects of chalcones to be applicable in the discovery of new drugs, pharmaceutical forms, using modern strategies, especially new nano-formulations in order to increase their bioavailability, prolonged effect, or transport to the target of the action. Further research and clinical trials can explore its pharmacological actions, their interactions with other compounds or medicines, and the level of toxicity it can cause.

## Author Contributions

JS-R, MM, and DC: conceptualization. BS, IC, NE, ABa, ABo, MA, and MI: validation investigation. CQ, JS-R, CC, AD, and MM: resources. CQ, JS-R, CC, GL-G, AD, MM, and FL: data curation. JS-R, AD, MM, DC, VL, and FL: review and editing. All authors: writing. All authors read and approved the final version, and contributed equally to the manuscript.

## Funding

This research and article processing charges were funded by a grant of Romanian Ministry of Research and Innovation, CCCDI-UEFISCDI project number 61PCCDI/2018 PN-III-P1-1.2-PCCDI-2017-0341, within PNCDI-III.

## Conflict of Interest

The authors declare that the research was conducted in the absence of any commercial or financial relationships that could be construed as a potential conflict of interest.

## References

[B1] Abbas A. Naseer M. M. Hasan A. Hadda T. B. (2014). Synthesis and cytotoxicity studies of 4-alkoxychalcones as new antitumor agents. J. Mater. Environ. Sci. 5, 281–292.

[B2] Acharjee S. Maity T. K. Samanta S. Mana S. Chakraborty T. Singha T. (2018). Antihyperglycemic activity of chalcone based novel 1-{3-[3-(substituted phenyl) prop-2-enoyl] phenyl} thioureas. Synth. Commun. 48, 3015–3024. 10.1080/00397911.2018.1539178

[B3] Ahmad Farooqi A. Fayyaz S. Silva A. S. Sureda A. Nabavi S. F. Mocan A. (2017). Oleuropein and cancer chemoprevention: the link is hot. Molecules 22, 705 10.3390/molecules22050705 PMC6154543 28468276

[B4] Ajaiyeoba E. Ogbole O. Abiodun O. Ashidi J. Houghton P. Wright C. W. (2013). Cajachalcone: an antimalarial compound from cajanus cajan leaf extract. J. Parasitol. Res. 2013, 703781 10.1155/2013/703781 23970954 PMC3732606

[B5] Alberton E. H. Damazio R. G. Cazarolli L. H. Chiaradia L. D. Leal P. C. Nunes R. J. (2008). Influence of chalcone analogues on serum glucose levels in hyperglycemic rats. Chem. Biol. Interact. 171, 355–362. 10.1016/j.cbi.2007.11.001 18164698

[B6] Amole K. L. Bello I. A. Oyewale A. O. (2019). Synthesis, characterization and antibacterial activities of new fluorinated chalcones. Chem. Afr. 2, 47–55. 10.1007/s42250-019-00043-4

[B7] Amolins M. W. Blagg B. (2009). Natural product inhibitors of Hsp90: potential leads for drug discovery. Mini Rev. Med. Chem. 9, 140–152. 10.2174/138955709787316056 19200020 PMC2659174

[B8] Andrighetti-Fröhner C. R. De Oliveira K. N. Gaspar-Silva D. Pacheco L. K. Joussef A. C. Steindel M. (2009). Synthesis, biological evaluation and SAR of sulfonamide 4-methoxychalcone derivatives with potential antileishmanial activity. Eur. J. Med. Chem. 44, 755–763. 10.1016/j.ejmech.2008.04.016 18554753

[B9] Ansari F. L. Umbreen S. Hussain L. Makhmoor T. Nawaz S. A. Lodhi M. A. (2005). Syntheses and biological activities of chalcone and 1, 5-benzothiazepine derivatives: promising new free-radical scavengers, and esterase, urease, and α-glucosidase inhibitors. Chem. Biodivers. 2, 487–496. 10.1002/cbdv.200590029 17191997

[B10] Araico A. Terencio M. Alcaraz M. Dominguez J. Leon C. Ferrandiz M. (2006). Phenylsulphonyl urenyl chalcone derivatives as dual inhibitors of cyclo-oxygenase-2 and 5-lipoxygenase. Life Sci. 78, 2911–2918. 10.1016/j.lfs.2005.11.017 16360707

[B11] Asogan M. V. Aupati V. R. (2016). Discovery of synthetic bioactive flavonoid derivatives as potential antidiabetic agents. Der Pharma Chem. 8, 152–168.

[B12] Attarde M. Vora A. Varghese A. Kachwala Y. (2014). Synthesis and evaluation of chalcone derivatives for its alpha amylase inhibitory activity. Org. Chem. An Indian J. 10, 192–204.

[B13] Avula B. Ganzera M. Warnick J. E. Feltenstein M. W. Sufka K. J. Khan I. A. (2004). High-performance liquid chromatographic determination of xanthohumol in rat plasma, urine, and fecal samples. J. Chromatogr. Sci. 42, 378–382. 10.1093/chromsci/42.7.378 15355578

[B14] Bak E. J. Park H. G. Lee C. Lee T.-I. Woo G.-H. Na Y. (2011). Effects of novel chalcone derivatives on α-glucosidase, dipeptidyl peptidase-4, and adipocyte differentiation *in vitro* . BMB Rep. 44, 410–414. 10.5483/BMBRep.2011.44.6.410 21699755

[B15] Bandgar B. P. Gawande S. S. (2010). Synthesis and biological screening of a combinatorial library of β-chlorovinyl chalcones as anticancer, anti-inflammatory and antimicrobial agents. Bioorg. Med. Chem. 18, 2060–2065. 10.1016/j.bmc.2009.12.077 20138527

[B16] Bandgar B. P. Patil S. A. Gacche R. N. Korbad B. L. Hote B. S. Kinkar S. N. (2010). Synthesis and biological evaluation of nitrogen-containing chalcones as possible anti-inflammatory and antioxidant agents. Bioorg. Med. Chem. Lett. 20, 730–733. 10.1016/j.bmcl.2009.11.068 20005707

[B17] Bano S. Javed K. Ahmad S. Rathish I. Singh S. Chaitanya M. (2013). Synthesis of some novel chalcones, flavanones and flavones and evaluation of their anti-inflammatory activity. Eur. J. Med. Chem. 65, 51–59. 10.1016/j.ejmech.2013.04.056 23693150

[B18] Batovska D. Slavova A. Bankova V. Tsvetkova I. Ninova M. Najdenski H. (2007). Study on the substituents' effects of a series of synthetic chalcones against the yeast candida albicans. Eur. J. Med. Chem. 42, 87–92. 10.1016/j.ejmech.2006.08.012 17007965

[B19] Bello M. L. Chiaradia L. D. Dias L. R. S. Pacheco L. K. Stumpf T. R. Mascarello A. (2011). Trimethoxy-chalcone derivatives inhibit growth of Leishmania braziliensis: synthesis, biological evaluation, molecular modeling and structure–activity relationship (SAR). Bioorg. Med. Chem. 19, 5046–5052. 10.1016/j.bmc.2011.06.023 21757358

[B20] Beltramino R. P. Penenory A. Buceta A. M. (1999). An open-label, randomised multicentre study comparing the efficacy and safety of CYCLO 3 FORT versus hydroxyethyl rutoside in chronic venous lymphatic insufficiency. Int. Angiol. 18, 337–342. 10811525

[B21] Beltramino R. Penenory A. Buceta A. M. (2000). An open-label, randomized multicenter study comparing the efficacy and safety of Cyclo 3 Fort® versus hydroxyethyl rutoside in chronic venous lymphatic insufficiency. Angiology 51, 535–544. 10.1177/000331970005100702 10917578

[B22] Bhakuni D. Chaturvedi R. (1984). Chemical constituents of Crotalaria madurensis. J. Nat. Prod. 47, 585–591. 10.1021/np50034a003 6491677

[B23] Bhale P. S. Dongare S. B. Chanshetti U. B. (2013). Synthesis and antimicrobial screening of chalcones containing imidazo [1, 2-a] pyridine nucleus. Res. J. Chem. Sci. 2231, 606.

[B24] Bharatham K. Bharatham N. Park K. H. Lee K. W. (2008). Binding mode analyses and pharmacophore model development for sulfonamide chalcone derivatives, a new class of α-glucosidase inhibitors. J. Mol. Graph. Model. 26, 1202–1212. 10.1016/j.jmgm.2007.11.002 18096420

[B25] Boccalon H. Causse C. Yubero L. (1998). Comparative efficacy of a single daily dose of two capsules Cyclo 3 Fort in the morning versus a repeated dose of one capsule morning and noon: a one-month study. Int. Angiol. 17, 155. 9821028

[B26] Boonchai W. Varothai S. Winayanuwattikun W. Phaitoonvatanakij S. Chaweekulrat P. Kasemsarn P. (2018). Randomized investigator-blinded comparative study of moisturizer containing 4-t-butylcyclohexanol and licochalcone A versus 0.02% triamcinolone acetonide cream in facial dermatitis. J. Cosmet. Dermatol. 17, 1130–1135. 10.1111/jocd.12499 29411520

[B27] Borges-Argaez R. Balnbury L. Flowers A. Giménez-Turba A. Ruiz G. Waterman P. G. (2007). Cytotoxic and antiprotozoal activity of flavonoids from Lonchocarpus spp. Phytomedicine 14, 530–533. 10.1016/j.phymed.2006.11.027 17291734

[B28] Boyle P. Diehm C. Robertson C. (2003). Meta-analysis of clinical trials of cyclo 3 Fort in the treatment of chronic venous insufficiency. Int. Angiol. 22, 250. 14612852

[B68] Brady S. Siegel G. Albers R. W. Price D. (2012). Basic Neurochemistry: Principles of Molecular, Cellular, and Medical Neurobiology. Elsevier Academic Press. Available at: 10.1016/C2009-0-00066-X

[B29] Burmaoglu S. Yilmaz A. O. Polat M. F. Kaya R. Gulcin I. Algul O. (2019). Synthesis and biological evaluation of novel tris-chalcones as potent carbonic anhydrase, acetylcholinesterase, butyrylcholinesterase and α-glycosidase inhibitors. Bioorg. Chem. 85, 191–197. 10.1016/j.bioorg.2018.12.035 30622011

[B30] Butler L. M. Ferraldeschi R. Armstrong H. K. Centenera M. M. Workman P. (2015). Maximizing the therapeutic potential of HSP90 inhibitors. Mol. Cancer Res. 13, 1445–1451. 10.1158/1541-7786.MCR-15-0234 26219697 PMC4645455

[B31] Cai C.-Y. Rao L. Rao Y. Guo J.-X. Xiao Z.-Z. Cao J.-Y. (2017). Analogues of xanthones—chalcones and bis-chalcones as α-glucosidase inhibitors and anti-diabetes candidates. Eur. J. Med. Chem. 130, 51–59. 10.1016/j.ejmech.2017.02.007 28242551

[B32] Calina D. Buga A. M. Mitroi M. Buha A. Caruntu C. Scheau C. (2020). The treatment of cognitive, behavioural and motor impairments from brain injury and neurodegenerative diseases through cannabinoid system modulation-evidence from *in vivo* studies. J. Clin. Med. 9 (8), 2395 10.3390/jcm9082395 PMC7464236 32726998

[B33] Cermak R. Durazzo A. Maiani G. Böhm V. Kammerer D. R. Carle R. (2009). The influence of postharvest processing and storage of foodstuffs on the bioavailability of flavonoids and phenolic acids. Mol. Nutr. Food Res. 53, S184–S193. 10.1002/mnfr.200700444 19035558

[B34] Chatsumpun N. Sritularak B. Likhitwitayawuid K. (2017). New biflavonoids with α-glucosidase and pancreatic lipase inhibitory activities from boesenbergia rotunda. Molecules 22, 1862 10.3390/molecules22111862 PMC6150212 29084164

[B35] Chatzopoulou M. Pegklidou K. Papastavrou N. Demopoulos V. J. (2013). Development of aldose reductase inhibitors for the treatment of inflammatory disorders. Expet Opin. Drug Discov. 8, 1365–1380. 10.1517/17460441.2013.843524 24090200

[B36] Chauhan S. S. Singh A. K. Meena S. Lohani M. Singh A. Arya R. K. (2014). Synthesis of novel β-carboline based chalcones with high cytotoxic activity against breast cancer cells. Bioorg. Med. Chem. Lett. 24, 2820–2824. 10.1016/j.bmcl.2014.04.109 24844196

[B37] Chen J.-J. Cheng M.-J. Shu C.-W. Sung P.-J. Lim Y.-P. Cheng L.-Y. (2017). A new chalcone and antioxidant constituents of Glycyrrhiza glabra. Chem. Nat. Compd. 53, 632–634. 10.1007/s10600-017-2077-1

[B38] Chen M. Christensen S. B. Blom J. Lemmich E. Nadelmann L. Fich K. (1993). Licochalcone A, a novel antiparasitic agent with potent activity against human pathogenic protozoan species of leishmania. Antimicrob. Agents Chemother. 37, 2550–2556. 10.1128/aac.37.12.2550 8109916 PMC192736

[B39] Chen M. Christensen S. Theander T. G. Kharazmi A. (1994). Antileishmanial activity of licochalcone A in mice infected with leishmania major and in hamsters infected with *Leishmania donovani* . Antimicrob. Agents Chemother. 38, 1339–1344. 10.1128/aac.38.6.1339 8092835 PMC188208

[B40] Chinthala Y. Thakur S. Tirunagari S. Chinde S. Domatti A. K. Arigari N. K. (2015). Synthesis, docking and ADMET studies of novel chalcone triazoles for anti-cancer and anti-diabetic activity. Eur. J. Med. Chem. 93, 564–573. 10.1016/j.ejmech.2015.02.027 25743216

[B41] Chu J. Guo C. L. (2016). Design and discovery of some novel chalcones as antioxidant and anti-inflammatory agents via attenuating NF-κB. Arch. Pharm. (Weinheim) 349, 63–70. 10.1002/ardp.201500349 26660296

[B42] Chularojanamontri L. Tuchinda P. Kulthanan K. Varothai S. Winayanuwattikun W. (2016). A double-blinded, randomized, vehicle-controlled study to access skin tolerability and efficacy of an anti-inflammatory moisturizer in treatment of acne with 0.1% adapalene gel. J. Dermatolog. Treat. 27, 140–145. 10.3109/09546634.2015.1079298 26293170

[B43] Daikonya A. Katsuki S. Kitanaka S. (2004). Antiallergic agents from natural sources 9. Inhibition of nitric oxide production by novel chalcone derivatives from Mallotus philippinensis (Euphorbiaceae). Chem. Pharm. Bull. (Tokyo) 52, 1326–1329. 10.1248/cpb.52.1326 15516755

[B44] Dal Picolo C. R. Bezerra M. P. Gomes K. S. Passero L. F. D. Laurenti M. D. Martins E. G. A. (2014). Antileishmanial activity evaluation of adunchalcone, a new prenylated dihydrochalcone from Piper aduncum L. Fitoterapia 97, 28–33. 10.1016/j.fitote.2014.05.009 24862066

[B45] Damazio R. G. Zanatta A. P. Cazarolli L. H. Chiaradia L. D. Mascarello A. Nunes R. J. (2010). Antihyperglycemic activity of naphthylchalcones. Eur. J. Med. Chem. 45, 1332–1337. 10.1016/j.ejmech.2009.12.017 20061067

[B46] Damazio R. G. Zanatta A. P. Cazarolli L. H. Mascarello A. Chiaradia L. D. Nunes R. J. (2009). Nitrochalcones: potential *in vivo* insulin secretagogues. Biochimie 91, 1493–1498. 10.1016/j.biochi.2009.09.002 19747522

[B47] De Mello T. F. Bitencourt H. R. Pedroso R. B. Aristides S. M. Lonardoni M. V. Silveira T. G. (2014). Leishmanicidal activity of synthetic chalcones in Leishmania (Viannia) braziliensis. Exp. Parasitol. 136, 27–34. 10.1016/j.exppara.2013.11.003 24269198

[B48] De R. V. Scambia G. Benedetti P. P. Ranelletti F. Bonanno G. Ercoli A. (1995). Effect of synthetic and naturally occurring chalcones on ovarian cancer cell growth: structure-activity relationships. Anticancer Drug Des. 10, 481–490. 7575989

[B49] Di L. Kerns E. H. Fan K. Mcconnell O. J. Carter G. T. (2003). High throughput artificial membrane permeability assay for blood–brain barrier. Eur. J. Med. Chem. 38, 223–232. 10.1016/s0223-5234(03)00012-6 12667689

[B50] Domínguez J. N. León C. Rodrigues J. De Domínguez N. G. Gut J. Rosenthal P. J. (2005). Synthesis and antimalarial activity of sulfonamide chalcone derivatives. Farmaco 60, 307–311. 10.1016/j.farmac.2005.01.005 15848205

[B51] Dominguez J. N. Leon C. Rodrigues J. De Dominguez N. G. Gut J. Rosenthal P. J. (2009). Synthesis of chlorovinyl sulfones as structural analogs of chalcones and their antiplasmodial activities. Eur. J. Med. Chem. 44, 1457–1462. 10.1016/j.ejmech.2008.09.044 19036479

[B52] Domínguez J. N. Charris J. E. Lobo G. De DomíNguez N. G. Moreno M. M. Riggione F. (2001). Synthesis of quinolinyl chalcones and evaluation of their antimalarial activity. Eur. J. Med. Chem. 36, 555–560. 10.1016/s0223-5234(01)01245-4 11525846

[B53] Dzoyem J. P. Hamamoto H. Ngameni B. Ngadjui B. T. Sekimizu K. (2013). Antimicrobial action mechanism of flavonoids from dorstenia species. Drug Discov. Ther. 7, 66–72. 10.5582/ddt.2013.v7.2.66 23715504

[B54] Elsohly H. Joshi A. Nimrod A. Walker L. Clark A. (2001). Antifungal chalcones from maclura tinctoria. Planta Med. 67, 87–89. 10.1055/s-2001-10621 11270732

[B55] Enoki T. Ohnogi H. Kobayashi E. Sagawa H. (2010). Anti-diabetic activities of chalcones derived from ashitaba. Nippon Shokuhin Kagaku Kogaku Kaishi 57, 456–463. 10.3136/nskkk.57.456

[B56] Enoki T. Ohnogi H. Nagamine K. Kudo Y. Sugiyama K. Tanabe M. (2007). Antidiabetic activities of chalcones isolated from a japanese herb, angelica keiskei. J. Agric. Food Chem. 55, 6013–6017. 10.1021/jf070720q 17583349

[B57] Farzaneh S. Zeinalzadeh E. Daraei B. Shahhosseini S. Zarghi A. (2018). New ferrocene compounds as selective cyclooxygenase (COX-2) inhibitors: design, synthesis, cytotoxicity and enzyme-inhibitory activity. Anticancer Agents Med. Chem. 18, 295–301. 10.2174/1871520617666171003145533 28971779

[B58] Ferlay J. Soerjomataram I. Ervik M. Dikshit R. Eser S. Mathers C. Rebelo M. Parkin D. M. Forman D. Bray F. (2013). GLOBOCAN 2012 v1.0, Cancer Incidence and Mortality Worldwide: IARC CancerBase No. 11 [Internet]. Lyon, France: International Agency for Research on Cancer. Available at: http://globocan.iarc.fr.

[B59] Filosa R. Peduto A. De Caprariis P. Saturnino C. Festa M. Petrella A. (2007). Synthesis and antiproliferative properties of N3/8-disubstituted 3, 8-diazabicyclo [3.2. 1] octane analogues of 3, 8-bis [2-(3, 4, 5-trimethoxyphenyl) pyridin-4-yl] methyl-piperazine. Eur. J. Med. Chem. 42, 293–306. 10.1016/j.ejmech.2006.11.013 17254669

[B60] Frölich S. Schubert C. Jenett-Siems K. (2009). Antimalarials from prenylated chalcone derivatives of hops. Beer Health Dis. Prev., 747–752. 10.1016/b978-0-12-373891-2.00075-4

[B61] Gabriela N. Rosa A. M. Catiana Z. I. Soledad C. Mabel O. R. Esteban S. J. (2014). The effect of zuccagnia punctata, an argentine medicinal plant, on virulence factors from candida species. Natural Prod. Commun. 9, 1934578X1400900712 10.1177/1934578x1400900712 25230496

[B62] Gaur R. Yadav K. S. Verma R. K. Yadav N. P. Bhakuni R. S. (2014). *In vivo* anti-diabetic activity of derivatives of isoliquiritigenin and liquiritigenin. Phytomedicine 21, 415–422. 10.1016/j.phymed.2013.10.015 24262065

[B63] Gerhäuser C. (2005). Beer constituents as potential cancer chemopreventive agents. Eur. J. Cancer 41, 1941–1954. 10.1016/j.ejca.2005.04.012 15953717

[B64] Geyer J. A. Keenan S. M. Woodard C. L. Thompson P. A. Gerena L. Nichols D. A. (2009). Selective inhibition of Pfmrk, a plasmodium falciparum CDK, by antimalarial 1, 3-diaryl-2-propenones. Bioorg. Med. Chem. Lett. 19, 1982–1985. 10.1016/j.bmcl.2009.02.042 19250824

[B65] Gil-Izquierdo A. Gil M. I. Ferreres F. Tomás-Barberán F. A. (2001). *In vitro* availability of flavonoids and other phenolics in orange juice. J. Agric. Food Chem. 49, 1035–1041. 10.1021/jf0000528 11262068

[B66] Gilmore T. D. (2006). Introduction to NF-κ B: players, pathways, perspectives. Oncogene 25, 6680–6684. 10.1038/sj.onc.1209954 17072321

[B67] Gomes M. N. Muratov E. N. Pereira M. Peixoto J. C. Rosseto L. P. Cravo P. V. (2017). Chalcone derivatives: promising starting points for drug design. Molecules 22, 1210 10.3390/molecules22081210 PMC6152227 28757583

[B69] Guantai E. M. Ncokazi K. Egan T. J. Gut J. Rosenthal P. J. Smith P. J. (2010). Design, synthesis and *in vitro* antimalarial evaluation of triazole-linked chalcone and dienone hybrid compounds. Bioorg. Med. Chem. 18, 8243–8256. 10.1016/j.bmc.2010.10.009 21044845

[B70] Gupta S. Shivahare R. Korthikunta V. Singh R. Gupta S. Tadigoppula N. (2014). Synthesis and biological evaluation of chalcones as potential antileishmanial agents. Eur. J. Med. Chem. 81, 359–366. 10.1016/j.ejmech.2014.05.034 24858541

[B71] Ha M. T. Seong S. H. Nguyen T. D. Cho W.-K. Ah K. J. Ma J. Y. (2018). Chalcone derivatives from the root bark of Morus alba L. act as inhibitors of PTP1B and α-glucosidase. Phytochemistry 155, 114–125. 10.1016/j.phytochem.2018.08.001 30103164

[B72] Hara H. Ikeda R. Ninomiya M. Kamiya T. Koketsu M. Adachi T. (2014). Newly synthesized ‘hidabeni’ chalcone derivatives potently suppress LPS-induced NO production via inhibition of STAT1, but not NF-κB, JNK, and p38, pathways in microglia. Biol. Pharm. Bull. 37, 1042–1049. 10.1248/bpb.b14-00116 24882415

[B73] Hayat F. Moseley E. Salahuddin A. Van Zyl R. L. Azam A. (2011). Antiprotozoal activity of chloroquinoline based chalcones. Eur. J. Med. Chem. 46, 1897–1905. 10.1016/j.ejmech.2011.02.004 21377771

[B74] Hermoso A. Jiménez I. A. Mamani Z. A. Bazzocchi I. L. Piñero J. E. Ravelo A. G. (2003). Antileishmanial activities of dihydrochalcones from piper elongatum and synthetic related compounds. structural requirements for activity. Bioorg. Med. Chem. 11, 3975–3980. 10.1016/s0968-0896(03)00406-1 12927858

[B75] Hideo K. Tatsurou H. (1997). Yoko and Kojima. Chem. Abstr. 126, 125

[B76] Huang Y. C. Guh J. H. Cheng Z. J. Chang Y. L. Hwang T. L. Lin C. N. (2001). Inhibitory effect of DCDC on lipopolysaccharide-induced nitric oxide synthesis in RAW 264.7 cells. Life Sci. 68, 2435–2447. 10.1016/s0024-3205(01)01035-9 11350014

[B77] Ianosi G. Ianosi S. Calbureanu-Popescu M. X. Tutunaru C. Calina D. Neagoe D. (2019). Comparative study in leg telangiectasias treatment with Nd: YAG laser and sclerotherapy. Exp. Ther. Med. 17, 1106–1112. 10.3892/etm.2018.6985 30679981 PMC6327418

[B78] Ianoşi S. Ianoşi G. Neagoe D. Ionescu O. Zlatian O. Docea A. O. (2016). Age-dependent endocrine disorders involved in the pathogenesis of refractory acne in women. Mol. Med. Rep. 14, 5501–5506. 10.3892/mmr.2016.5924 27840992 PMC5355698

[B79] Ianosi S. Neagoe D. Branisteanu D. E. Popescu M. Calina D. Zlatian O. (2018). Comparative efficacy of oral contraceptive versus local treatment versus intense pulsed light combined with vacuum in endocrine acne in women. J. Biol. Regul. Homeost. Agents 32, 711–718. 29921404

[B80] Imran S. Taha M. Ismail N. H. Kashif S. M. Rahim F. Jamil W. (2015). Synthesis of novel flavone hydrazones: in-vitro evaluation of α-glucosidase inhibition, QSAR analysis and docking studies. Eur. J. Med. Chem. 105, 156–170. 10.1016/j.ejmech.2015.10.017 26491979

[B81] Jadhav S. Y. Bhosale R. B. Shirame S. P. Hublikar M. G. Sonawane K. D. Shaikh R. V. (2013). Synthesis and biological evaluation of fluoro-hydroxy substituted pyrazole chalcones as anti-inflammatory, antioxidant and antibacterial agents. Int. J. Pharm. Biol. Sci. 4, 309–397.

[B82] Jain A. Jain D. (2017). Docking, synthesis and evaluation of novel derivatives of substituted chalcones as antihyperglycemic agents. J. Drug Deliv. Ther. 7, 154–157.

[B83] Jamal H. Ansari W. Rizvi S. (2009). Chalcones: differential effects on glycogen contents of liver, brain, and spinal cord in rats. Biol. Med. 1, 107–115.

[B84] Jantan I. Bukhari S. N. A. Adekoya O. A. Sylte I. (2014). Studies of synthetic chalcone derivatives as potential inhibitors of secretory phospholipase A2, cyclooxygenases, lipoxygenase and pro-inflammatory cytokines. Drug Des. Devel. Ther. 8, 1405 10.2147/DDDT.S67370 PMC4172049 25258510

[B85] Jayanthi M. Jegatheesan K. Vidhya R. Kanagavalli U. (2012). Hypoglycemic effect of 2-hydroxychalcone on high fructose fed diabetic rat. Int. J. Pharm. Sci. Res. 3, 600.

[B86] Jayasinghe L. Balasooriya B. Padmini W. C. Hara N. Fujimoto Y. (2004). Geranyl chalcone derivatives with antifungal and radical scavenging properties from the leaves of artocarpus nobilis. Phytochemistry 65, 1287–1290. 10.1016/j.phytochem.2004.03.033 15184014

[B87] Jeon K.-H. Yu H.-B. Kwak S. Y. Kwon Y. Na Y. (2016). Synthesis and topoisomerases inhibitory activity of heteroaromatic chalcones. Bioorg. Med. Chem. 24, 5921–5928. 10.1016/j.bmc.2016.09.051 27707625

[B88] Jeong C.-H. Park H. B. Jang W. J. Jung S. H. Seo Y. H. (2014). Discovery of hybrid Hsp90 inhibitors and their anti-neoplastic effects against gefitinib-resistant non-small cell lung cancer (NSCLC). Bioorg. Med. Chem. Lett. 24, 224–227. 10.1016/j.bmcl.2013.11.034 24345447

[B89] Kakkos S. K. Bouskela E. Jawien A. Nicolaides A. N. (2018). New data on chronic venous disease: a new place for cyclo 3® fort. Int. Angiol. 37, 85–92. 10.23736/S0392-9590.17.03935-9 29063748

[B90] Kim D. H. Li H. Han Y. E. Jeong J. H. Lee H. J. Ryu J.-H. (2018). Modulation of inducible nitric oxide synthase expression in LPS-stimulated BV-2 microglia by prenylated chalcones from Cullen corylifolium (L.) Medik. Through inhibition of I-κBα degradation. Molecules 23, 109 10.3390/molecules23010109 PMC6017879 29300354

[B91] Kim D. U. Chung H. C. Kim C. Hwang J. K. (2017). Oral intake of boesenbergia pandurata extract improves skin hydration, gloss, and wrinkling: a randomized, double-blind, and placebo-controlled study. J. Cosmet. Dermatol. 16, 512–519. 10.1111/jocd.12343 28421656

[B92] Kim J. H. Ryu Y. B. Kang N. S. Lee B. W. Heo J. S. Jeong I.-Y. (2006). Glycosidase inhibitory flavonoids from sophora flavescens. Biol. Pharm. Bull. 29, 302–305. 10.1248/bpb.29.302 16462036

[B93] Kim M. J. Kadayat T. Da Eun Kim E.-S. L. Park P.-H. (2014). TI-I-174, a synthetic chalcone derivative, suppresses nitric oxide production in murine macrophages via heme oxygenase-1 induction and inhibition of AP-1. Biomol. Ther. (Seoul) 22, 390 10.4062/biomolther.2014.062 25414768 PMC4201222

[B94] Kim Y. H. Kim J. Park H. Kim H. P. (2007). Anti-inflammatory activity of the synthetic chalcone derivatives: inhibition of inducible nitric oxide synthase-catalyzed nitric oxide production from lipopolysaccharide-treated RAW 264.7 cells. Biol. Pharm. Bull. 30, 1450–1455. 10.1248/bpb.30.1450 17666802

[B95] Kolbe L. Immeyer J. Batzer J. Wensorra U. Tom Dieck K. Mundt C. (2006). Anti-inflammatory efficacy of licochalcone A: correlation of clinical potency and *in vitro* effects. Arch. Dermatol. Res. 298, 23–30. 10.1007/s00403-006-0654-4 16552540

[B96] Koudokpon H. Armstrong N. Dougnon T. Fah L. Hounsa E. Bankolé H. (2018). Antibacterial activity of chalcone and dihydrochalcone compounds from uvaria chamae roots against multidrug-resistant bacteria. Biomed Res. Int. 2018, 1453173 10.1155/2018/1453173 30225246 PMC6129846

[B97] Kucerova-Chlupacova M. Kunes J. Buchta V. Vejsova M. Opletalova V. (2015). Novel pyrazine analogs of chalcones: synthesis and evaluation of their antifungal and antimycobacterial activity. Molecules 20, 1104–1117. 10.3390/molecules20011104 25587786 PMC6272410

[B98] Kulkarni R. R. Tupe S. G. Gample S. P. Chandgude M. G. Sarkar D. Deshpande M. V. (2014). Antifungal dimeric chalcone derivative kamalachalcone E from mallotus philippinensis. Nat. Prod. Res. 28, 245–250. 10.1080/14786419.2013.843178 24099509

[B99] Kumar S. Sharma A. Madan B. Singhal V. Ghosh B. (2007). Isoliquiritigenin inhibits IκB kinase activity and ROS generation to block TNF-α induced expression of cell adhesion molecules on human endothelial cells. Biochem. Pharmacol. 73, 1602–1612. 10.1016/j.bcp.2007.01.015 17276410

[B100] Lee M. H. Kim J. Y. Ryu J. H. (2005). Prenylflavones from psoralea corylifolia inhibit nitric oxide synthase expression through the inhibition of I-kappaB-alpha degradation in activated microglial cells. Biol. Pharm. Bull. 28, 2253–2257. 10.1248/bpb.28.2253 16327160

[B101] Li R. Kenyon G. L. Cohen F. E. Chen X. Gong B. Dominguez J. N. (1995). *In vitro* antimalarial activity of chalcones and their derivatives. J. Med. Chem. 38, 5031–5037. 10.1021/jm00026a010 8544179

[B102] Liaras K. Geronikaki A. Glamočlija J. Ćirić A. Soković M. (2011). Thiazole-based chalcones as potent antimicrobial agents. synthesis and biological evaluation. Bioorg. Med. Chem. 19, 3135–3140. 10.1016/j.bmc.2011.04.007 21524583

[B103] Lichota A. Gwozdzinski L. Gwozdzinski K. (2019). Therapeutic potential of natural compounds in inflammation and chronic venous insufficiency. Eur. J. Med. Chem. 176, 68–91. 10.1016/j.ejmech.2019.04.075 31096120

[B104] Lima T. C. Souza R. J. Santos A. D. Moraes M. H. Biondo N. E. Barison A. (2016). Evaluation of leishmanicidal and trypanocidal activities of phenolic compounds from calea uniflora less. Nat. Prod. Res. 30, 551–557. 10.1080/14786419.2015.1030740 25880257

[B105] Liu M. Wilairat P. Croft S. L. Tan A. L.-C. Go M.-L. (2003). Structure–activity relationships of antileishmanial and antimalarial chalcones. Bioorg. Med. Chem. 11, 2729–2738. 10.1016/s0968-0896(03)00233-5 12788347

[B106] Liu M. Yin H. Liu G. Dong J. Qian Z. Miao J. (2014). Xanthohumol, a prenylated chalcone from beer hops, acts as an α-glucosidase inhibitor *in vitro* . J. Agric. Food Chem. 62, 5548–5554. 10.1021/jf500426z 24897556

[B107] Lopez S. N. Castelli M. V. Zacchino S. A. DomíNguez J. N. Lobo G. Charris-Charris J. (2001). *In vitro* antifungal evaluation and structure–activity relationships of a new series of chalcone derivatives and synthetic analogues, with inhibitory properties against polymers of the fungal cell wall. Bioorg. Med. Chem. 9, 1999–2013. 10.1016/s0968-0896(01)00116-x 11504637

[B108] Maccari R. Ottanà R. (2015). Targeting aldose reductase for the treatment of diabetes complications and inflammatory diseases: new insights and future directions. J. Med. Chem. 58, 2047–2067. 10.1021/jm500907a 25375908

[B109] Mahapatra D. K. Bharti S. K. Asati V. (2017a). Chalcone derivatives: anti-inflammatory potential and molecular targets perspectives. Curr. Top. Med. Chem. 17, 3146–3169. 10.2174/1568026617666170914160446 28914193

[B110] Mahapatra D. K. Chhajed S. S. Shivhare R. S. (2017b). Development of Murrayanine-Chalcone hybrids: an effort to combine two privilege scaffolds for enhancing hypoglycemic activity. Int. J. Pharm. Chem. Anal. 4, 30–34.

[B111] Mellado M. Espinoza L. Madrid A. Mella J. Chávez-Weisser E. Diaz K. (2019). Design, synthesis, antifungal activity, and structure–activity relationship studies of chalcones and hybrid dihydrochromane–chalcones. Mol. Divers. 24 (3), 603–615. 10.1007/s11030-019-09967-y 31161394

[B112] Mititelu R. R. Pădureanu R. Băcănoiu M. Pădureanu V. Docea A. O. Calina D. (2020). Inflammatory and oxidative stress markers—mirror tools in rheumatoid arthritis. Biomedicines 8, 125 10.3390/biomedicines8050125 PMC7277871 32429264

[B113] Mocan A. Vlase L. Vodnar D. C. Bischin C. Hanganu D. Gheldiu A.-M. (2014). Polyphenolic content, antioxidant and antimicrobial activities of Lycium barbarum L. and Lycium chinense Mill. leaves. Molecules 19, 10056–10073. 10.3390/molecules190710056 25014533 PMC6271913

[B114] Monisha E. Suganya V. Anuradha V. Syed Ali M. (2018). Antioxidant, anti-inflammatory and antidiabetic activity of some novel chalcone and piperidine derivatives. Int. Res. J. Pharmacy Med. Sci. 2, 2581–3277.

[B115] Moosavi M. A. Haghi A. Rahmati M. Taniguchi H. Mocan A. Echeverría J. (2018). Phytochemicals as potent modulators of autophagy for cancer therapy. Cancer Lett. 424, 46–69. 10.1016/j.canlet.2018.02.030 29474859

[B116] Mounika K. L. S. (2015). Silico evaluation of alpha glucosidase and alpha amylase, inhibitory activity of chemical constituents from psoralea corylifolia. Int. J. Chem. Res. 8, 532–538.

[B117] Naidu M. A. Prasad Y. R. (2018). Evaluation of antidiabetic activity of novel diarylsulfonylureachalcone hybrids in streptozotocin-induced diabetic models in rats. *Asian J. Pharm* . 12, S382‐S405. 10.22377/ajp.v12i01.2464

[B118] Najafian M. Ebrahim-Habibi A. Yaghmaei P. Parivar K. Larijani B. (2010). Core structure of flavonoids precursor as an antihyperglycemic and antihyperlipidemic agent: an *in vivo* study in rats. Acta Biochim. Pol. 57, 553–560. 10.18388/abp.2010_2443 21060897

[B119] Ngameni B. Watchueng J. Boyom F. F. Keumedjio F. Ngadjui B. T. Gut J. (2007). Antimalarial prenylated chalcones from the twigs of dorstenia barteri var. subtriangularis. Arkivoc 13, 116–123. 10.3998/ark.5550190.0008.d14

[B120] Nowakowska Z. (2007). A review of anti-infective and anti-inflammatory chalcones. Eur. J. Med. Chem. 42, 125–137. 10.1016/j.ejmech.2006.09.019 17112640

[B121] Nyandoro S. Nkunya M. Josepha C. Odalo J. Sattler I. (2012). New glucopyranosylglyceryl-N-octenyl adipate and bioactivity of retro and condensed chalcones from Toussaintia orientalis. Tanzan. J. Sci. 38, 108–126.

[B122] Oh Y. J. Seo Y. H. (2017). A novel chalcone-based molecule, BDP inhibits MDA-MB-231 triple-negative breast cancer cell growth by suppressing Hsp90 function. Oncol. Rep. 38, 2343–2350. 10.3892/or.2017.5925 28849241

[B123] Ohta M. Fujinami A. Kobayashi N. Amano A. Ishigami A. Tokuda H. (2015). Two chalcones, 4-hydroxyderricin and xanthoangelol, stimulate GLUT4-dependent glucose uptake through the LKB1/AMP-activated protein kinase signaling pathway in 3T3-L1 adipocytes. Nutr. Res. 35, 618–625. 10.1016/j.nutres.2015.05.010 26077869

[B124] Okuda-Tanino A. Sugawara D. Tashiro T. Iwashita M. Obara Y. Moriya T. (2017). Licochalcones extracted from Glycyrrhiza inflata inhibit platelet aggregation accompanied by inhibition of COX-1 activity. PLoS One 12, e0173628 10.1371/journal.pone.0173628 28282426 PMC5345862

[B125] Orlikova B. Schnekenburger M. Zloh M. Golais F. Diederich M. Tasdemir D. (2012). Natural chalcones as dual inhibitors of HDACs and NF-κB. Oncol. Rep. 28, 797–805. 10.3892/or.2012.1870 22710558 PMC3583578

[B126] Otero E. Vergara S. Robledo S. M. Cardona W. Carda M. Vélez I. D. (2014). Synthesis, leishmanicidal and cytotoxic activity of triclosan-chalcone, triclosan-chromone and triclosan-coumarin hybrids. Molecules 19, 13251–13266. 10.3390/molecules190913251 25170948 PMC6271011

[B127] Özdemir A. Altıntop M. D. Turan-Zitouni G. Çiftçi G. A. Ertorun I. Alataş Ö. (2015). Synthesis and evaluation of new indole-based chalcones as potential antiinflammatory agents. Eur. J. Med. Chem. 89, 304–309. 10.1016/j.ejmech.2014.10.056 25462246

[B128] Padureanu R. Albu C. V. Mititelu R. R. Bacanoiu M. V. Docea A. O. Calina D. (2019). Oxidative stress and inflammation interdependence in multiple sclerosis. J. Clin. Med. 8, 1815 10.3390/jcm8111815 PMC6912446 31683787

[B129] Pajouhesh H. Lenz G. R. (2005). Medicinal chemical properties of successful central nervous system drugs. NeuroRx 2, 541–553. 10.1602/neurorx.2.4.541 16489364 PMC1201314

[B130] Passalacqua T. G. Torres F. A. Nogueira C. T. De Almeida L. Del Cistia M. L. Dos Santos M. B. (2015). The 2′, 4′-dihydroxychalcone could be explored to develop new inhibitors against the glycerol-3-phosphate dehydrogenase from leishmania species. Bioorg. Med. Chem. Lett. 25, 3564–3568. 10.1016/j.bmcl.2015.06.085 26169126

[B131] Peralta G. A. Gardoqui J. A. Macías F. L. Ceja V. N. Cisneros S. M. Macías C. M. (2007). Clinical and capillaroscopic evaluation in the treatment of chronic venous insufficiency with Ruscus aculeatus, hesperidin methylchalcone and ascorbic acid in venous insufficiency treatment of ambulatory patients. Int. Angiol. 26, 378. 18091707

[B132] Prasath R. Bhavana P. Ng S. W. Tiekink E. R. (2013). The facile and efficient ultrasound-assisted synthesis of new quinoline-appended ferrocenyl chalcones and their properties. J. Organomet. Chem. 726, 62–70. 10.1016/j.jorganchem.2012.12.022

[B133] Purnima S. Beena P. Mini R. Swaminathan P. (2012). Study of the effect of Adhatoda zeylanica and some related synthesized chalcones on glucose diffusion *in vitro* . ARPB 2, 259–263.

[B134] Rahaman S. Prasad Y. R. Bhuvaneswari K. Kumar P. (2010). Synthesis and antihistaminic activity of novel pyrazoline derivatives. Int. J. Chem. Tech. Res. 2, 16–20.

[B135] Raju D. B. Rao A. V. Prasad Y. R. (2018). Hybrid sulfonylurea-linked chalconoids as antidiabetic agents: evaluation of antihyperglycemic effects in streptozotocin-induced type 2 diabetic rats. Rasayan J. Chem. 11, 1334–1338. 10.31788/rjc.2018.1133084

[B136] Rao M. V. Rao A. V. Mujavar E. (2014). Evaluation of the *in vivo* hypoglycemic effect of sulfonylurea-chalcone hybrid molecules in normoglycemic rabbits. J. Global Trends Pharm. Sci. 5, 1797–1803.

[B137] Rawat P. Kumar M. Rahuja N. Srivastava D. S. L. Srivastava A. K. Maurya R. (2011). Synthesis and antihyperglycemic activity of phenolic C-glycosides. Bioorg. Med. Chem. Lett 21, 228–233. 10.1016/j.bmcl.2010.11.031 21129962

[B138] Real L. (1967). Duvud С and Francois В. J. Pharmacol. Sci. 2, 37.

[B139] Ribnicky D. M. Kuhn P. Poulev A. Logendra S. Zuberi A. Cefalu W. T. (2009). Improved absorption and bioactivity of active compounds from an anti-diabetic extract of artemisia dracunculus L. Int. J. Pharm. 370, 87–92. 10.1016/j.ijpharm.2008.11.012 19084584 PMC3130197

[B140] Rocha S. Sousa A. Ribeiro D. Correia C. M. Silva V. L. Santos C. M. (2019). A study towards drug discovery for the management of type 2 diabetes mellitus through inhibition of the carbohydrate-hydrolyzing enzymes α-amylase and α-glucosidase by chalcone derivatives. Food Funct. 10, 5510–5520. 10.1039/c9fo90045d 31414099

[B141] Rojas J. DomíNguez J. N. Charris J. E. Lobo G. Payá M. Ferrándiz M. L. (2002). Synthesis and inhibitory activity of dimethylamino-chalcone derivatives on the induction of nitric oxide synthase. Eur. J. Med. Chem. 37, 699–705. 10.1016/s0223-5234(02)01387-9 12161067

[B142] Rojas J. Payá M. Devesa I. Dominguez J. N. Ferrándiz M. L. (2003a). Therapeutic administration of 3, 4, 5-trimethoxy-4'-fluorochalcone, a selective inhibitor of iNOS expression, attenuates the development of adjuvant-induced arthritis in rats. Naunyn Schmiedebergs Arch. Pharmacol. 368, 225–233. 10.1007/s00210-003-0780-x 12904830

[B143] Rojas J. Payá M. DomíNguez J. N. Ferrándiz M. L. (2003b). ttCH, a selective inhibitor of inducible nitric oxide synthase expression with antiarthritic properties. Eur. J. Pharmacol. 465, 183–189. 10.1016/s0014-2999(03)01457-2 12650848

[B144] Romagnoli R. Baraldi P. G. Carrion M. D. Cara C. L. Cruz-Lopez O. Preti D. (2008). Design, synthesis, and biological evaluation of thiophene analogues of chalcones. Bioorg. Med. Chem. 16, 5367–5376. 10.1016/j.bmc.2008.04.026 18440234

[B145] Ryu H. W. Lee B. W. Curtis-Long M. J. Jung S. Ryu Y. B. Lee W. S. (2010). Polyphenols from Broussonetia papyrifera displaying potent α-glucosidase inhibition. J. Agric. Food Chem. 58, 202–208. 10.1021/jf903068k 19954213

[B146] Salehi B. Capanoglu E. Adrar N. Catalkaya G. Shaheen S. Jaffer M. (2019a). Cucurbits plants: a key emphasis to its pharmacological potential. Molecules 24, 1854 10.3390/molecules24101854 PMC6572650 31091784

[B147] Salehi B. Lopez-Jornet P. Pons-Fuster López E. Calina D. Sharifi-Rad M. Ramírez-Alarcón K. (2019b). Plant-derived bioactives in oral mucosal lesions: a key emphasis to curcumin, lycopene, chamomile, aloe vera, green tea and coffee properties. Biomolecules 9, 106 10.3390/biom9030106 PMC6468600 30884918

[B148] Salehi B. Sestito S. Rapposelli S. Peron G. Calina D. Sharifi-Rad M. (2019c). Epibatidine: a promising natural alkaloid in health. Biomolecules 9, 6 10.3390/biom9010006 PMC6359223 30583611

[B149] Salehi B. Sharifi-Rad J. Capanoglu E. Adrar N. Catalkaya G. Shaheen S. (2019d). Cucurbita plants: from farm to industry. Appl. Sci. 9, 3387 10.3390/app9163387

[B150] Salehi B. Shivaprasad Shetty M. Anil Kumar V. N. Živković J. Calina D. (2019e). Veronica plants—drifting from farm to traditional healing, food application, and phytopharmacology. Molecules 24, 2454 10.3390/molecules24132454 PMC6651156 31277407

[B151] Salehi B. Calina D. Docea A. O. Koirala N. Aryal S. Lombardo D. (2020a). Curcumin’s nanomedicine formulations for therapeutic application in neurological diseases. J. Clin. Med. 9, 430 10.3390/jcm9020430 PMC7074182 32033365

[B152] Salehi B. Rescigno A. Dettori T. Calina D. Docea A. O. Singh L. (2020b). Avocado–Soybean unsaponifiables: a panoply of potentialities to Be exploited. Biomolecules 10, 130 10.3390/biom10010130 PMC7023362 31940989

[B153] Salehi B. Sharifi-Rad J. Cappellini F. Reiner Ž. Zorzan D. Imran M. (2020c). The therapeutic potential of anthocyanins: current approaches based on their molecular mechanism of action. Front. Pharmacol. 11, 1300 10.3389/fphar.2020.01300 32982731 PMC7479177

[B154] Salem M. M. Werbovetz K. A. (2005). Antiprotozoal compounds from Psorothamnus p olydenius. J. Nat. Prod. 68, 108–111. 10.1021/np049682k 15679330

[B155] Salem M. M. Werbovetz K. A. (2006). Isoflavonoids and other compounds from Psorothamnus a rborescens with antiprotozoal activities. J. Nat. Prod. 69, 43–49. 10.1021/np049682k 16441066

[B156] Satyanarayana M. Tiwari P. Tripathi B. K. Srivastava A. Pratap R. (2004). Synthesis and antihyperglycemic activity of chalcone based aryloxypropanolamines. Bioorg. Med. Chem. 12, 883–889. 10.1016/j.bmc.2003.12.026 14980600

[B157] Scheau C. Badarau I. A. Mihai L.-G. Scheau A.-E. Costache D. O. Constantin C. (2020). Cannabinoids in the pathophysiology of skin inflammation. Molecules 25, 652 10.3390/molecules25030652 PMC7037408 32033005

[B158] Schroder J. (1999). The chalcone/stibene synthase-type family of condensing enzymes. Compr. Nat. Prod. Chem. 1, 749–771. 10.1016/b978-0-08-091283-7.00029-1

[B159] Schweiger D. Baufeld C. Drescher P. Oltrogge B. Höpfner S. Mess A. (2013). Efficacy of a new tonic containing urea, lactate, polidocanol, and glycyrrhiza inflata root extract in the treatment of a dry, itchy, and subclinically inflamed scalp. Skin Pharmacol. Physiol. 26, 108–118. 10.1159/000348473 23549137

[B160] Semwal D. K. Rawat U. Semwal R. Singh R. Krishan P. Singh M. (2009). Chemical constituents from the leaves of Boehmeria rugulosa with antidiabetic and antimicrobial activities. J. Asian Nat. Prod. Res. 11, 1045–1055. 10.1080/10286020903352526 20183275

[B161] Sengupta S. A. Maity T. K. Samanta S. (2017). Synthesis, biological screening and in silico studies of chalcone based novel phenyl urea derivatives as potential antihyperglycemics. J. Pharm. Res. 16, 237–246. 10.18579/jpcrkc/2017/16/3/118765

[B162] Seo W. D. Kim J. H. Kang J. E. Ryu H. W. Curtis-Long M. J. Lee H. S. (2005). Sulfonamide chalcone as a new class of α-glucosidase inhibitors. Bioorg. Med. Chem. Lett. 15, 5514–5516. 10.1016/j.bmcl.2005.08.087 16202584

[B163] Sharifi-Rad J. Kamiloglu S. Yeskaliyeva B. Beyatli A. Alfred M. A. Salehi B. (2020a). Pharmacological activities of Psoralidin: a comprehensive review of the molecular mechanisms of action. Front. Pharmacol. 11, 571459 10.3389/fphar.2020.571459 33192514 PMC7643726

[B164] Sharifi-Rad J. Rodrigues C. F. Sharopov F. Docea A. O. Can Karaca A. Sharifi-Rad M. (2020b). Diet, lifestyle and cardiovascular diseases: linking pathophysiology to cardioprotective effects of natural bioactive compounds. Int. J. Environ. Res. Public Health 17, 2326 10.3390/ijerph17072326 PMC7177934 32235611

[B165] Sharifi-Rad M. Anil Kumar N. V. Zucca P. Varoni E. M. Dini L. Panzarini E. (2020c). Lifestyle, oxidative stress, and antioxidants: back and Forth in the pathophysiology of chronic diseases. Front. Physiol. 11, 694 10.3389/fphys.2020.00694 32714204 PMC7347016

[B166] Sharifi-Rad M. Lankatillake C. Dias D. A. Docea A. O. Mahomoodally M. F. Lobine D. (2020d). Impact of natural compounds on neurodegenerative disorders: from preclinical to pharmacotherapeutics. J. Clin. Med. 9, 1061 10.3390/jcm9041061 PMC7231062 32276438

[B167] Sharifi-Rad M. Lankatillake C. Dias D. A. Docea A. O. Mahomoodally M. F. Lobine D. (2020e). Impact of natural compounds on neurodegenerative disorders: from preclinical to pharmacotherapeutics. J. Clin. Med. 9, 1061 10.3390/jcm9041061 PMC7231062 32276438

[B168] Shibata S. (1994). Anti-tumorigenic chalcones. Stem cells 12, 44–52. 10.1002/stem.5530120109 8142919

[B169] Shimokoriyama M. (1962). Flavanones, chalcones and aurones. In: T. Geissman (ed.) The Chemistry of Flavonoid Compounds. New York: The Macmillan Co.

[B170] Shin J. Jang M. G. Park J. C. Do Koo Y. Lee J. Y. Park K. S. (2018). Antidiabetic effects of trihydroxychalcone derivatives via activation of AMP-activated protein kinase. J. Ind. Eng. Chem. 60, 177–184. 10.1016/j.jiec.2017.11.003

[B171] Shirley B. W. (1996). Flavonoid biosynthesis:‘new’functions for an ‘old’pathway. Trends Plant Sci. 1, 377–382.

[B172] Shukla P. Satyanarayana M. Verma P. C. Tiwari J. Dwivedi A. P. Srivastava R. (2017). Chalcone-based aryloxypropanolamine as a potential antidiabetic and antidyslipidaemic agent. Curr. Sci. 112, 1675–1689. 10.18520/cs/v112/i08/1675-1689

[B173] Shukla P. Singh A. B. Srivastava A. K. Pratap R. (2007). Chalcone based aryloxypropanolamines as potential antihyperglycemic agents. Bioorg. Med. Chem. Lett. 17, 799–802. 10.1016/j.bmcl.2006.10.068 17095211

[B174] Siddiqui Z. N. Praveen S. Musthafa T. M. Ahmad A. Khan A. U. (2012). Thermal solvent-free synthesis of chromonyl chalcones, pyrazolines and their *in vitro* antibacterial, antifungal activities. J. Enzym. Inhib. Med. Chem. 27, 84–91. 10.3109/14756366.2011.577035 21612378

[B175] Sifaki M. Calina D. Docea A. O. Tsioumas S. Katsarou M. S. Papadogiorgaki S. (2020). A novel approach regarding the anti-aging of facial skin through collagen reorganization. Exp. Ther. Med. 19, 717–721. 10.3892/etm.2019.8254 31885709 PMC6913239

[B176] Singh A. Fong G. Liu J. Wu Y.-H. Chang K. Park W. (2018). Synthesis and preliminary antimicrobial analysis of isatin–ferrocene and isatin–ferrocenyl chalcone conjugates. ACS Omega 3, 5808–5813. 10.1021/acsomega.8b00553 30023926 PMC6045481

[B177] Sousa-Batista A. D. J. Philipon C. I. S. De Souza Albernaz M. Pinto S. R. Rossi-Bergmann B. Santos-Oliveira R. (2018). New chalcone compound as a promising antileishmanial drug for an old neglected disease: biological evaluation using radiolabelled biodistribution. J. Glob. Antimicrob. Resist. 13, 139–142. 10.1016/j.jgar.2017.11.012 29196220

[B178] Sulzberger M. Worthmann A. C. Holtzmann U. Buck B. Jung K. Schoelermann A. (2016). Effective treatment for sensitive skin: 4-t-butylcyclohexanol and licochalcone A. J. Eur. Acad. Dermatol. Venereol. 30, 9–17. 10.1111/jdv.13529 26805417

[B179] Sun H. Li Y. Zhang X. Lei Y. Ding W. Zhao X. (2015). Synthesis, α-glucosidase inhibitory and molecular docking studies of prenylated and geranylated flavones, isoflavones and chalcones. Bioorg. Med. Chem. Lett. 25, 4567–4571. 10.1016/j.bmcl.2015.08.059 26351039

[B180] Sun H. Wang D. Song X. Zhang Y. Ding W. Peng X. (2017). Natural prenylchalconaringenins and prenylnaringenins as antidiabetic agents: α-glucosidase and α-amylase inhibition and *in vivo* antihyperglycemic and antihyperlipidemic effects. J. Agric. Food Chem. 65, 1574–1581. 10.1021/acs.jafc.6b05445 28132506

[B181] Svetaz L. Tapia A. López S. N. Furlán R. L. Petenatti E. Pioli R. (2004). Antifungal chalcones and new caffeic acid esters from Zuccagnia punctata acting against soybean infecting fungi. J. Agric. Food Chem. 52, 3297–3300. 10.1021/jf035213x 15161186

[B182] Syam S. Abdelwahab S. I. Al-Mamary M. A. Mohan S. (2012). Synthesis of chalcones with anticancer activities. Molecules 17, 6179–6195. 10.3390/molecules17066179 22634834 PMC6268294

[B183] Tajammal A. Batool M. Ramzan A. Samra M. M. Mahnoor I. Verpoort F. (2017). Synthesis, antihyperglycemic activity and computational studies of antioxidant chalcones and flavanones derived from 2, 5 dihydroxyacetophenone. J. Mol. Struct. 1148, 512–520. 10.1016/j.molstruc.2017.07.042

[B184] Tajuddeen N. Isah M. B. Suleiman M. A. Van Heerden F. R. Ibrahim M. A. (2018). The chemotherapeutic potential of chalcones against leishmaniases: a review. Int. J. Antimicrob. Agents 51, 311–318. 10.1016/j.ijantimicag.2017.06.010 28668673

[B185] Takahashi M. Maeda S. Ogura K. Terano A. Omata M. (1998). The possible role of vascular endothelial growth factor (VEGF) in gastric ulcer healing: effect of sofalcone on VEGF release *in vitro* . J. Clin. Gastroenterol. 27, S178–S182. 10.1097/00004836-199800001-00029 9872518

[B186] Tan R. X. Meng J. C. Hostettmann K. (2000). Phytochemical investigation of some traditional chinese medicines and endophyte cultures. Pharm. Biol. 38, 25–32. 10.1076/phbi.38.6.25.5955 23531135

[B187] Tang C. Zhu L. Chen Y. Qin R. Mei Z. Xu J. (2014). Synthesis and biological evaluation of oleanolic acid derivative–chalcone conjugates as α-glucosidase inhibitors. RSC Adv. 4, 10862–10874. 10.1039/c3ra46492j

[B188] Toiu A. Mocan A. Vlase L. Pârvu A. E. Vodnar D. C. Gheldiu A.-M. (2019). Comparative phytochemical profile, antioxidant, antimicrobial and *in vivo* anti-inflammatory activity of different extracts of traditionally used romanian ajuga genevensis L. and A. reptans L. (Lamiaceae). Molecules 24, 1597 10.3390/molecules24081597 PMC6515068 31018502

[B189] Tomar V. Bhattacharjee G. Kumar A. (2007). Synthesis and antimicrobial evaluation of new chalcones containing piperazine or 2, 5-dichlorothiophene moiety. Bioorg. Med. Chem. Lett. 17, 5321–5324. 10.1016/j.bmcl.2007.08.021 17719779

[B190] Tomar V. Bhattacharjee G. Rajakumar S. Srivastava K. Puri S. (2010). Synthesis of new chalcone derivatives containing acridinyl moiety with potential antimalarial activity. Eur. J. Med. Chem. 45, 745–751. 10.1016/j.ejmech.2009.11.022 20022412

[B191] Tomás-Barberán F. A. Clifford M. N. (2000). Flavanones, chalcones and dihydrochalcones–nature, occurrence and dietary burden. J. Sci. Food Agric. 80, 1073–1080. 10.1002/(sici)1097-0010(20000515)80:7<1073::aid-jsfa568>3.0.co;2-b

[B192] Torres-Santos E. C. Moreira D. L. Kaplan M. a. C. Meirelles M. N. Rossi-Bergmann B. (1999). Selective effect of 2′, 6′-dihydroxy-4′-methoxychalcone isolated from Piper aduncum on *Leishmania amazonensis* . Antimicrob. Agents Chemother. 43, 1234–1241. 10.1128/AAC.43.5.1234 10223942 PMC89139

[B193] Tsatsakis, A. Docea A. O. Calina D. Tsarouhas K. Zamfira L.-M. Mitrut R. (2019). A mechanistic and pathophysiological approach for stroke associated with drugs of abuse. J. Clin. Med. 8, 1295 10.3390/jcm8091295 PMC6780697 31450861

[B194] Tsatsakis, A. M. Docea A. O. Calina D. Buga A. M. Zlatian O. Gutnikov S. (2019). Hormetic Neurobehavioral effects of low dose toxic chemical mixtures in real-life risk simulation (RLRS) in rats. Food Chem. Toxicol. 125, 141–149. 10.1016/j.fct.2018.12.043 30594548

[B195] Udompataikul M. Srisatwaja W. (2011). Comparative trial of moisturizer containing licochalcone A vs. hydrocortisone lotion in the treatment of childhood atopic dermatitis: a pilot study. J. Eur. Acad. Dermatol. Venereol. 25, 660–665. 10.1111/j.1468-3083.2010.03845.x 20840345

[B196] Ugwu D. I. Ezema B. E. Okoro U. C. Eze F. U. Ekoh O. C. Egbujor M. C. (2015). Syntheses and pharmacological applications of chalcones: a review. Int. J. Chem. Sci. 13, 459–500.

[B197] Ungureanu A. Zlatian O. Mitroi G. Drocaş A. Ţîrcă T. Călina D. (2017). *Staphylococcus aureus* colonisation in patients from a primary regional hospital. Mol. Med. Rep. 16, 8771–8780. 10.3892/mmr.2017.7746 29039613 PMC5779955

[B198] Vijaya Bhaskar Reddy M. Hung H.-Y. Kuo P.-C. Huang G.-J. Chan Y.-Y. Huang S.-C. (2017). Synthesis and biological evaluation of chalcone, dihydrochalcone, and 1,3-diarylpropane analogs as anti-inflammatory agents. Bioorg. Med. Chem. Lett. 27, 1547–1550. 10.1016/j.bmcl.2017.02.038 28256373

[B199] Viveka S. Naik P. Nagaraja G. K. (2014). Synthesis, characterization of new imidazoquinonyl chalcones and pyrazolines as potential anticancer and antioxidant agents. Med. Chem. Res. 23, 4189–4197. 10.1007/s00044-014-0998-9

[B200] Wananukul S. Chatproedprai S. Chunharas A. Limpongsanuruk W. Singalavanija S. Nitiyarom R. (2013). Randomized, double-blind, split-side, comparison study of moisturizer containing licochalcone A and 1% hydrocortisone in the treatment of childhood atopic dermatitis. J. Med. Assoc. Thai. 96, 1135–1142. 24163988

[B201] Watanabe Y. Nagai Y. Honda H. Okamoto N. Yamamoto S. Hamashima T. (2016). Isoliquiritigenin attenuates adipose tissue inflammation *in vitro* and adipose tissue fibrosis through inhibition of innate immune responses in mice. Sci. Rep. 6, 23097 10.1038/srep23097 26975571 PMC4791553

[B202] Weber T. Ceilley R. Buerger A. Kolbe L. Trookman N. Rizer R. (2006). Skin tolerance, efficacy, and quality of life of patients with red facial skin using a skin care regimen containing licochalcone A. J. Cosmet. Dermatol. 5, 227–232. 10.1111/j.1473-2165.2006.00261.x 17177744

[B203] Weindorf N. Schultz-Ehrenburg U. (1987). Controlled study of increasing venous tone in primary varicose veins by oral administration of Ruscus aculeatus and trimethylhespiridinchalcone. Z. Hautkr. 62, 28–38. 3554800

[B204] Won S.-J. Liu C.-T. Tsao L.-T. Weng J.-R. Ko H.-H. Wang J.-P. (2005). Synthetic chalcones as potential anti-inflammatory and cancer chemopreventive agents. Eur. J. Med. Chem. 40, 103–112. 10.1016/j.ejmech.2004.09.006 15642415

[B205] Yadav N. Dixit S. K. Bhattacharya A. Mishra L. C. Sharma M. Awasthi S. K. (2012). Antimalarial activity of newly synthesized chalcone derivatives *in vitro* . Chem. Biol. Drug Des. 80, 340–347. 10.1111/j.1747-0285.2012.01383.x 22429524

[B206] Yin B.-T. Yan C.-Y. Peng X.-M. Zhang S.-L. Rasheed S. Geng R.-X. (2014). Synthesis and biological evaluation of α-triazolyl chalcones as a new type of potential antimicrobial agents and their interaction with calf thymus DNA and human serum albumin. Eur. J. Med. Chem. 71, 148–159. 10.1016/j.ejmech.2013.11.003 24291568

[B207] Zajac M. Muszalska I. Jelinska A. (2016). New molecular targets of anticancer therapy–current status and perspectives. Curr. Med. Chem. 23, 4176–4220. 10.2174/0929867323666160814002150 27528054

[B208] Zhang L. Lu Y. (2012). Inhibitory activities of extracts from Cleistocalyx operculatus flower buds on pancreatic lipase and α-amylase. Eur. Food Res. Technol. 235, 1133–1139. 10.1248/bpb.26.61

[B209] Zhao F. Nozawa H. Daikonnya A. Kondo K. Kitanaka S. (2003). Inhibitors of nitric oxide production from hops (Humulus lupulus L.). Biol. Pharm. Bull. 26, 61–65. 10.1248/bpb.26.61 12520174

[B210] Zhu P. Huang W. Li J. Ma X. Hu M. Wang Y. (2018). Design, synthesis chalcone derivatives as AdipoR agonist for type 2 diabetes. Chem. Biol. Drug Des. 92, 1525–1536. 10.1111/cbdd.13319 29704399

[B211] Zhuang C. Zhang W. Sheng C. Zhang W. Xing C. Miao Z. (2017). Chalcone: a privileged structure in medicinal chemistry. Chem. Rev. 117, 7762–7810. 10.1021/acs.chemrev.7b00020 28488435 PMC6131713

[B212] Žiberna L. Šamec D. Mocan A. Nabavi S. F. Bishayee A. Farooqi A. A. (2017). Oleanolic acid alters multiple cell signaling pathways: implication in cancer prevention and therapy. Int. J. Mol. Sci. 18, 643 10.3390/ijms18030643 PMC5372655 28300756

[B213] Zuzarte M. Vale-Silva L. Gonçalves M. Cavaleiro C. Vaz S. Canhoto J. (2012). Antifungal activity of phenolic-rich Lavandula multifida L. essential oil. Eur. J. Clin. Microbiol. Infect. Dis. 31, 1359–1366. 10.1007/s10096-011-1450-4 22020493

